# The global burden of cancer attributable to dietary factors from 1990 to 2019

**DOI:** 10.1017/S1368980024002489

**Published:** 2025-01-09

**Authors:** Jiping Xie, Jing Zhao

**Affiliations:** Department of Oncology, Yuyao People’s Hospital, Ningbo 315400, Zhejiang, China

**Keywords:** Cancer, Dietary factors, Global burden of disease, Estimated annual percentage change, Sociomorphic index

## Abstract

**Objective:**

To analyze the global cancer burden associated with dietary factors across 204 countries and regions from 1990 to 2019.

**Design:**

A population-based study.

**Setting:**

Global Burden of Disease Study.

**Participants:**

Using data from the 2019 global burden of disease, we calculated Population Attributable Fractions (PAFs), death and disability-adjusted life years (DALYs). A comparative risk assessment framework was employed, along with estimated annual percentage changes (EAPCs).

**Results:**

In 2019, approximately 6·01 % of cancer mortality and 5·50 % of DALY rates can be attributed to dietary risk factors, particularly low intake of whole grains, milk, and fruits and vegetables. The High Socio-Demographic Index (SDI) region had the highest cancer mortality and DALY PAFs, mainly due to high consumption of red and processed meats, while the Low SDI region showed the highest PAFs from low fruit and vegetable consumption. In 2019, the High-middle SDI region had the highest age-standardized death rate (ASDR) and DALY rate attributable to dietary factors. Among geographic regions, Southern Latin America had the highest ASDR, and Central Europe had the highest age-standardized DALY rate. At the country level, Mongolia exhibited the highest rates for both ASDR and DALYs attributable to dietary risks. From 1990 to 2019, the largest increase in ASDR was observed in Western Sub-Saharan Africa, with Bulgaria showing the largest country-specific increase. Similarly, the largest increase in the age-standardized DALY rate was seen in Western Sub-Saharan Africa, with Lesotho experiencing the highest increase at the country level.

**Conclusions:**

Our findings underscored the importance of increasing the consumption of whole grains, milk, and calcium, which can inform global dietary guidelines and cancer prevention strategies.

Cancer is the second leading cause of death worldwide, following CVD, with approximately 18·1 million new cancer cases and nearly 10 million cancer-related deaths reported in 2020^(1)^. According to a 2014 study, environmental factors are responsible for 95 % of cancer cases, with genetic factors contributing only 5 %^(2)^. It is important to note that more recent research may present slightly different estimates, but the significant impact of environmental factors remains a critical consideration. Dietary factors emerge as particularly significant within the environmental category, rivalling and even surpassing traditional risk factors such as smoking in certain contexts. For example, while smoking is widely recognized as a leading risk factor for lung and other cancers^(3)^, dietary influences often exceed smoking’s impact in terms of attributable cases of colorectal, breast, and prostate cancers^(4)^. This shift underscoreds the growing importance of dietary considerations in cancer prevention strategies globally. Developed countries attribute around 30 % of cancer cases to dietary factors, while developing countries estimate this impact to be around 20 %^(5)^. Current evidence highlights the potential for preventing and reducing cancer incidence by modifying these factors.

Over the past three decades, the influence of diet on cancer development and prevention has grown due to the rise in unhealthy food consumption driven by commercial interests^(6)^. The World Cancer Research Fund and the American Institute for Cancer Research (WCRF/AICR) have published comprehensive reports indicating strong associations between certain dietary patterns and cancer risk^(7)^. High consumption of red and processed meats has been linked to an increased risk of colorectal cancer, with processed meat classified as a Group 1 carcinogen by the International Agency for Research on Cancer (IARC)^(8)^. Conversely, diets rich in fruits, vegetables, and whole grains are associated with a reduced risk of several cancers, including those of the mouth, pharynx, larynx, esophagus, and stomach^(9,10)^. Dairy products and calcium intake have been shown to influence the risk of colorectal cancer, potentially due to calcium’s protective effect on the mucosal lining of the colon^(11,12)^. Additionally, vitamin D, obtained from diet and sunlight exposure, may play a role in reducing cancer risk through its effects on cell growth regulation^(13)^. Despite this growing body of evidence, variations in dietary habits across different regions make it challenging to quantify the specific contribution of dietary factors to the global cancer burden. This underscoreds the necessity of gaining a deeper understanding of dietary risk factors in cancer incidence and addressing geographical disparities in cancer distribution.

This study aimed to explore the current understanding of the association between dietary factors and cancer risk. Utilizing the 2019 Global Burden of Disease (GBD) data, the analysis involved stratification by sex, age, and Socio-Demographic Index (SDI) values. Through the assessment of mortality rates, disability-adjusted life years, and age-standardized rates, the objective was to examine the changing patterns of cancer burden associated with dietary factors across 204 countries and regions from 1990 to 2019.

The ultimate goal of this research is to identify and analyze the impact of dietary factors on cancer burden, thereby providing scientific evidence to inform stakeholders in the development of effective cancer prevention strategies.

## Methods

### Data sources

The data utilized in this study is sourced from the 2019 GDB database, which collected demographic and epidemiological information on quantifiable cancers attributed to dietary factors across 204 countries and 21 regions between 1990 and 2019^(14)^. The dataset includes figures on deaths, disability adjusted life years, and age standardized rates. It is important to note that this research did not involve ethical approval or informed consent, as it falls under public data access.

### Cancer estimation

The Cause of Death (COD) database compiles various sources of cancer mortality data, such as vital registration, verbal autopsy, and cancer registration data. The cancer registry mortality estimates in the COD database are derived from cancer registry incidence data that is converted into mortality estimates using mortality to incidence rates (MIR). Prior to integration with the COD database, the cancer registration data undergoes several processing steps, the details of which have been documented and published elsewhere^(15)^.

According to the 10th edition of the International Classification of Diseases (ICD-10), there are 28 cancer groups that can be classified, which includes tumor-related ICD codes (ICD-9140-239; ICD-10, C00-D49), with the exception of Kaposi’s sarcoma (KS) (C46) and non-melanoma skin cancer (NMSC) (C44)^(16)^.

### Disability-adjusted life years (DALY)

DALY generally refers to the total number of years of healthy life lost from onset to death. This includes years of life lost due to premature death (YLL) and years of healthy life lost due to disability (YLD).

### SDI

The SDI serves as a comprehensive indicator of a country/region’s development status. It is assessed based on factors such as the overall fertility rate of women under 25 years old, the average education level of women aged 15 and above, and per capita income. The SDI values range from 0 to 1, with higher values indicating a higher level of development^(17)^. The index is categorized into five stages: Low SDI, Low middle SDI, Middle SDI, High middle SDI, and High SDI.

### Selection of dietary risk factors

The dietary data used in GBD is sourced from the Food and Agriculture Organization’s Food Balance Table and the Global Nutrition Database. The GBD2019 database compiles consumption data for each dietary factor from across the globe^(18)^. The 9 risk factors identified in the GBD Comparative Risk Assessment (CRA) framework are linked to cancer outcomes. These risk factors include diets low in fruits, vegetables, whole grains, and milk, as well as diets high in red and processed meats. Additionally, diets low in fiber and calcium are also considered. The data for these dietary risks (excluding diets high in sodium) is obtained from the 24-hour dietary recall survey, which records food and nutrient intake in grams per person per day. Sodium intake data, on the other hand, is collected from 24-hour urine sodium concentration^(19)^.

### Statistical analysis

Initially, the theoretical minimum risk exposure level is identified to determine the Population Attributable Fractions (PAFs) by comparing it with specific population exposure levels. Following the 2019 GDB comparative risk assessment framework, the cancer burden associated with dietary factors is calculated by multiplying PAFs with the overall cancer burden for each age, sex, and region. The annual estimated percentage change (EAPC) is commonly utilized to indicate the trend of rate changes over a defined period.

To compute the age-standardized mortality rate and DALY rate attributed to dietary risk factors between 1990 and 2019, we employed a generalized linear regression model with Gaussian analysis, using direct standardization. The Estimated Annual Percentage Changes (EAPC) and their 95 % CIs were calculated to assess trends. If both the EAPC and the lower limit of the 95 % CI are positive, the age-standardized rate (ASR) displays an upward trend; if they are negative, ASR shows a decreasing trend; otherwise, ASR remains stable^(20)^. Consequently, a 95 % CI (CI) for the age-standardized rate per 100 000 is obtained. All statistical analyses were performed using R version 4·1·3.

## Result

### Global trends in cancer burden attributed to dietary risk factors between 1990 and 2019

In 2019, 6·01 % of cancer mortality rates (95 % uncertainty interval (UI): 8·11, 4·56) and 5·55 % of cancer disability-adjusted life years (95 % UI: 7·53, 4·21) could be attributed to dietary risk factors measured in the GBD study. The global number of cancer deaths linked to dietary factors rose from 372 355·76 in 1990 to 605 427·05 in 2019, while the DALY attributable to dietary factors increased from 9 368 997·08 in 1990 to 13 951 293·26 in 2019. In 2019, the age-standardized death rate (ASDR) and age-standardized DALY rate attributable to dietary factors for cancer were 7·56 and 168·77 per 100 000 individuals, respectively, with ASDR (EAPC = –1·02) and age-standardized DALY rate (EAPC = –1·16) exhibiting a downward trend from 1990 to 2019. Among the 9 cancer risk factors analyzed, the three factors with the most significant impact on cancer ASDR and age-standardized DALY rate from 1990 to 2019 were a diet low in whole grains, a diet low in milk, and a diet rich in antioxidants, fiber, and phytochemicals (such as fruits, vegetables, and legumes).

### Global trends considering variations by sex and age

**Figure 1.**
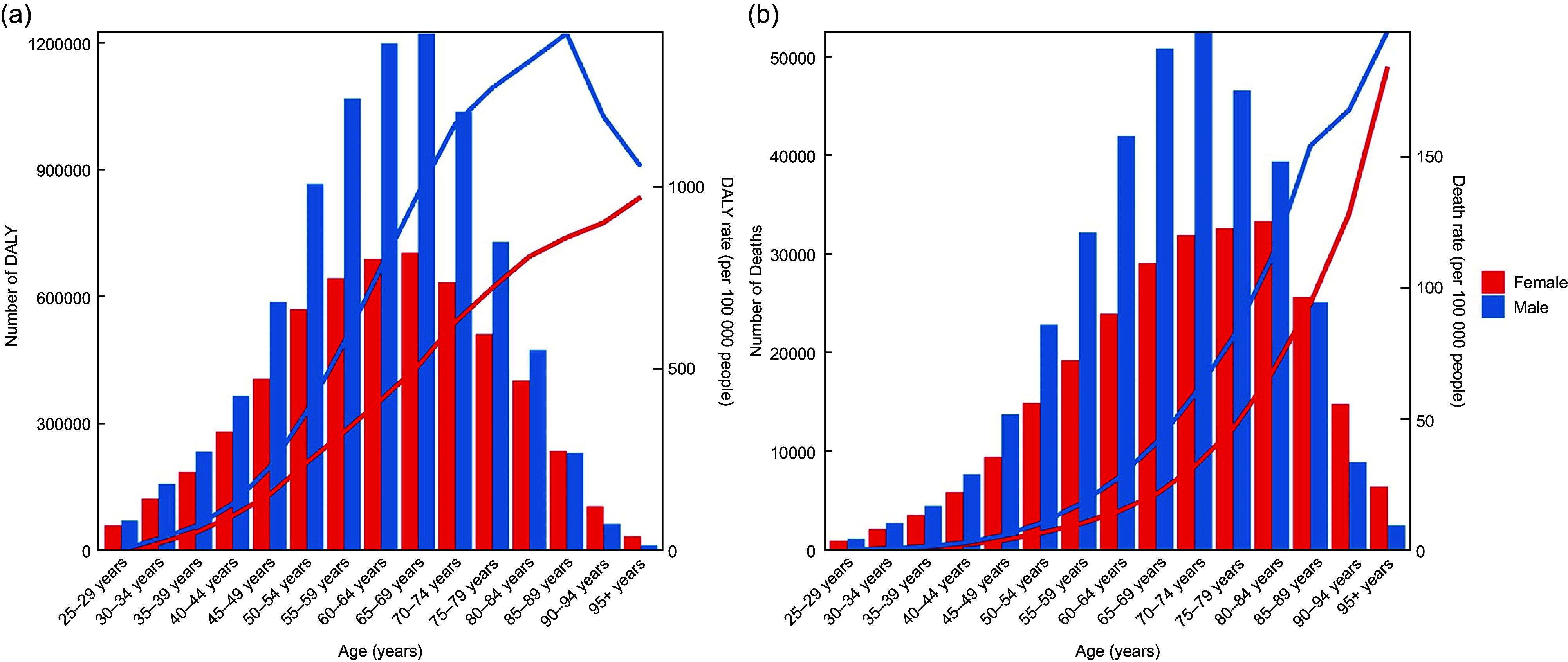
Age-specific numbers and rates of deaths and DALYs attributable to dietary factors by age, by sex, in 2019. (a) DALYs. (b) Death. DALY, disability-adjusted life year.

In 2019, the impact of dietary risk factors on cancer deaths and DALYs varied by age and sex. Cancer mortality and DALY rates due to dietary risks were higher in males than females, peaking between the ages of 65 and 69 for both sexs. Males under 80 years old had notably higher DALYs than females, while females over 80 had more than males. As shown in Fig. 1, the DALY rate for cancer attributable to dietary factors increases with age for those under 85, but decreases for men over 85. The highest number of deaths linked to these factors occurred in males aged 70–74 and females aged 80–84. In individuals younger than 85, cancer deaths were more common in males, but in those older than 85, females had more deaths than males. The cancer mortality rate related to dietary factors was higher in males and increased significantly with age.

### Cancer burden attributable to dietary risk factors by SDI region

**Figure 2.**
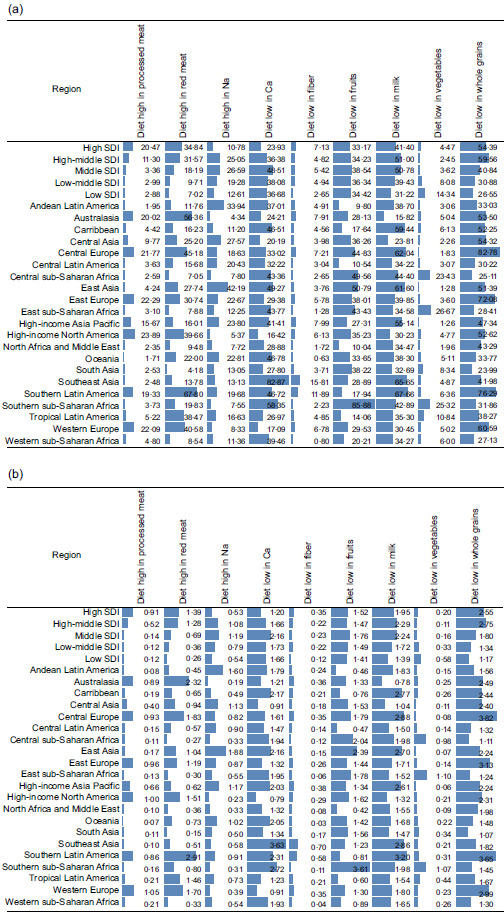
Proportion of death and DALYs attributable to dietary risk factors in 2019. (a) DALYs. (b) Death. DALY, disability-adjusted life year; SDI, sociodemographic index.

**Figure 3.**
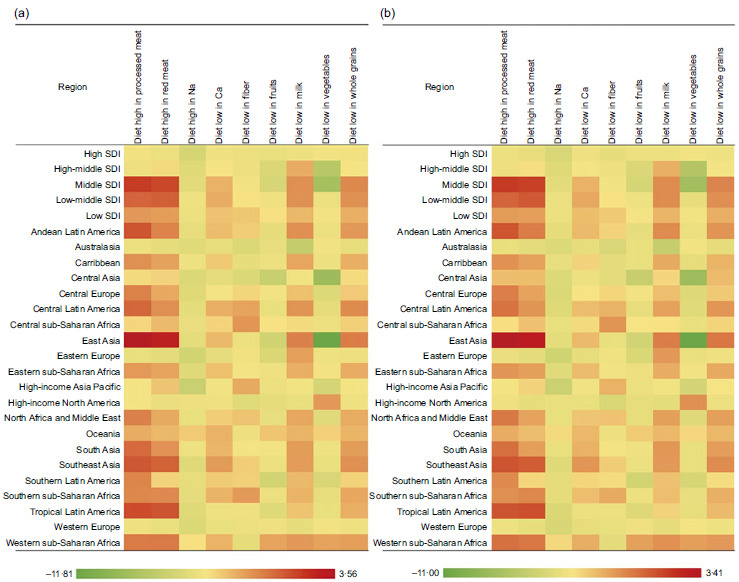
The age-standardized rate estimated annual percent change, 1990–2019. (a) DALYs. (b) Death. DALY, disability-adjusted life year; SDI, sociodemographic index.

In 2019, the ASDR and age-standardized DALY rates were highest in the High middle SDI region (8·34 and 188·73 per 100 000 people, respectively) attributable to dietary risk factors. This was followed by the high SDI regions with rates of 7·90 and 172·63, and the lowest rates were recorded in the Low SDI regions at 5·38 and 125·92. The ASDR and age-standardized DALY rates of cancer attributable to dietary risk factors exhibited a decreasing trend across all SDI regions from 1990 to 2019 (EAPC < 0). The largest decrease in ASDR was observed in the High SDI regions, while the age standardized DALY rate showed the largest decrease in the High middle SDI region, with smaller decreases in the Low SDI and Low middle SDI regions. In 2019, cancer deaths and DALYs attributable to high red and processed meat intake were higher in high SDI regions, whereas deaths and DALYs attributable to low vegetable intake were higher in low SDI regions. Furthermore, in cancer deaths and life loss attributable to dietary risk factors, high sodium, low calcium, and low fruit intake contributed to a higher proportion of total PAFs in the medium SDI region compared to the high and low SDI regions (Figs 2 and 3).

### Regional distribution of cancer burden

In 2019, among the 21 GBD regions, Southern Latin America (Argentina, Chile and Uruguay) (10·89) had the highest cancer-related ASDR attributable to dietary factors for every 100 000 people, followed by Central Europe (10·32) and Southern Sub-Saharan Africa (9·42). The total PAF of cancer deaths caused by dietary risk factors in Southern Latin America is the highest (7·23). On the other hand, the regions with the lowest ASDR are North Africa and Middle East (4·26), Central Latin America (4·78), and South Asia (4·83), respectively. Noteworthy changes in ASDREAPC attributable to dietary risk factors from 1990 to 2019 were observed in Western Sub-Saharan Africa (increase of 0·69) and Central Asia (decrease of –1·70). Additionally, Bulgaria showed the largest increase (188 %) and Turkey the largest decrease (323 %) in ASDREAPC. In 2019, among the 21 GBD regions, the highest age standardized DALY rate for cancer attributable to dietary factors per 100 000 people was found in Central Europe (235·03), Southern Latin America (234·86), Southern Sub-Saharan Africa (216·43), and High-income Asia Pacific (248·03), respectively. The total PAF for cancer DALYs due to dietary risk factors was 6·78. Conversely, North Africa and Middle East (96·57), Western Sub-Saharan Africa (108·00), and Central Latin America (110·01) had the lowest age standardized DALY rates for cancer caused by dietary factors. Notable changes in age standardized DALY rate EAPC attributable to dietary risk factors from 1990 to 2019 were identified in Western Sub-Saharan Africa (increase of 0·53) and Central Asia (decrease of –2·16). Furthermore, Lesotho experienced the highest increase (171 %) and Turkey the highest decrease (339 %) in age standardized DALY rate EAPC (Table 1, see online supplementary material, Supplemental Tables S1 and S2, Fig. 2).

**Table 1.** In twenty-one regions, cancer death/disability caused by dietary risk factors resulted in the total PAF value of adjusted life year (95 % uncertainty interval)

		1990	1990	1990	1990	1990	1990	2019	2019	2019	2019	2019	2019
		Deaths	Deaths	Deaths	DALYs	DALYs	DALYs	Deaths	Deaths	Deaths	DALYs	DALYs	DALYs
	Sex	Val	Upper	Lower	Val	Upper	Lower	Val	Upper	Lower	Val	Upper	Lower
Global	Both	0·06	0·09	0·05	0·06	0·08	0·04	0·06	0·08	0·05	0·06	0·08	0·04
High-middle SDI	Both	0·07	0·10	0·05	0·06	0·09	0·04	0·06	0·09	0·05	0·06	0·08	0·04
High SDI	Both	0·07	0·09	0·05	0·06	0·08	0·05	0·06	0·07	0·05	0·06	0·07	0·05
Low-middle SDI	Both	0·05	0·08	0·04	0·04	0·07	0·03	0·06	0·08	0·04	0·05	0·07	0·04
Low SDI	Both	0·05	0·07	0·03	0·04	0·06	0·03	0·05	0·07	0·04	0·04	0·05	0·03
Middle SDI	Both	0·07	0·11	0·04	0·06	0·09	0·04	0·06	0·09	0·04	0·06	0·08	0·04
Andean Latin America	Both	0·05	0·10	0·03	0·04	0·08	0·02	0·05	0·09	0·03	0·04	0·08	0·03
Australasia	Both	0·07	0·09	0·06	0·07	0·08	0·06	0·06	0·08	0·05	0·06	0·07	0·05
Caribbean	Both	0·05	0·07	0·04	0·04	0·06	0·03	0·05	0·06	0·04	0·04	0·06	0·03
Central Asia	Both	0·08	0·12	0·05	0·07	0·10	0·05	0·05	0·08	0·04	0·05	0·08	0·04
Central Europe	Both	0·06	0·09	0·04	0·06	0·08	0·04	0·06	0·08	0·05	0·06	0·07	0·05
Central Latin America	Both	0·04	0·08	0·03	0·03	0·06	0·02	0·05	0·07	0·03	0·04	0·07	0·03
Central sub-Saharan Africa	Both	0·05	0·07	0·04	0·04	0·06	0·03	0·05	0·07	0·04	0·05	0·06	0·03
East Asia	Both	0·08	0·13	0·05	0·07	0·11	0·04	0·07	0·10	0·04	0·06	0·10	0·04
Eastern Europe	Both	0·07	0·11	0·05	0·07	0·11	0·05	0·07	0·09	0·05	0·06	0·08	0·05
Eastern Sub-Saharan Africa	Both	0·05	0·07	0·04	0·04	0·06	0·02	0·05	0·07	0·04	0·04	0·05	0·03
High-income Asia Pacific	Both	0·07	0·13	0·05	0·07	0·12	0·04	0·07	0·10	0·05	0·07	0·10	0·05
High-income North America	Both	0·06	0·07	0·05	0·06	0·07	0·04	0·05	0·06	0·04	0·05	0·06	0·04
North Africa and Middle East	Both	0·04	0·06	0·03	0·03	0·05	0·02	0·04	0·05	0·03	0·04	0·05	0·03
Oceania	Both	0·05	0·07	0·03	0·04	0·06	0·03	0·05	0·07	0·04	0·04	0·06	0·03
South Asia	Both	0·05	0·08	0·04	0·04	0·06	0·03	0·05	0·07	0·04	0·04	0·06	0·03
Southeast Asia	Both	0·06	0·08	0·05	0·05	0·07	0·04	0·07	0·08	0·06	0·06	0·08	0·05
Southern Latin America	Both	0·07	0·09	0·05	0·06	0·08	0·05	0·07	0·09	0·06	0·07	0·08	0·05
Southern sub-Saharan Africa	Both	0·07	0·09	0·05	0·07	0·08	0·05	0·07	0·08	0·05	0·06	0·08	0·05
Tropical Latin America	Both	0·05	0·08	0·03	0·04	0·07	0·03	0·05	0·07	0·04	0·05	0·07	0·04
Western Europe	Both	0·06	0·08	0·05	0·06	0·08	0·05	0·06	0·07	0·05	0·06	0·07	0·05
Western sub-Saharan Africa	Both	0·04	0·07	0·03	0·03	0·05	0·03	0·04	0·06	0·03	0·04	0·05	0·03
global	Female	0·06	0·09	0·05	0·05	0·08	0·04	0·06	0·08	0·04	0·05	0·07	0·04
High-middle SDI	Female	0·07	0·10	0·05	0·06	0·09	0·04	0·06	0·08	0·05	0·06	0·07	0·04
High SDI	Female	0·07	0·09	0·05	0·06	0·08	0·05	0·06	0·07	0·05	0·06	0·07	0·05
Low-middle SDI	Female	0·05	0·08	0·04	0·04	0·06	0·03	0·05	0·07	0·04	0·04	0·06	0·03
Low SDI	Female	0·04	0·06	0·03	0·03	0·05	0·02	0·04	0·06	0·03	0·03	0·05	0·03
Middle SDI	Female	0·06	0·10	0·04	0·05	0·08	0·04	0·06	0·08	0·04	0·05	0·07	0·04
Andean Latin America	Female	0·04	0·09	0·03	0·03	0·07	0·02	0·05	0·08	0·03	0·04	0·07	0·03
Australasia	Female	0·08	0·09	0·06	0·07	0·09	0·06	0·07	0·08	0·05	0·07	0·08	0·05
Caribbean	Female	0·05	0·06	0·04	0·04	0·05	0·03	0·05	0·06	0·04	0·04	0·05	0·03
Central Asia	Female	0·07	0·11	0·05	0·06	0·09	0·05	0·05	0·07	0·04	0·05	0·07	0·03
Central Europe	Female	0·06	0·08	0·04	0·05	0·07	0·04	0·06	0·08	0·05	0·06	0·07	0·04
Central Latin America	Female	0·04	0·07	0·03	0·03	0·05	0·02	0·05	0·07	0·03	0·04	0·06	0·03
Central sub-Saharan Africa	Female	0·04	0·06	0·03	0·03	0·05	0·02	0·04	0·06	0·03	0·04	0·05	0·03
East Asia	Female	0·07	0·12	0·05	0·06	0·10	0·04	0·06	0·09	0·04	0·06	0·09	0·04
Eastern Europe	Female	0·08	0·11	0·06	0·07	0·10	0·05	0·07	0·09	0·05	0·06	0·08	0·04
Eastern Sub-Saharan Africa	Female	0·05	0·07	0·03	0·03	0·05	0·02	0·05	0·06	0·04	0·04	0·05	0·03
High-income Asia Pacific	Female	0·07	0·12	0·05	0·07	0·11	0·04	0·07	0·10	0·05	0·07	0·09	0·05
High-income North America	Female	0·06	0·08	0·05	0·06	0·07	0·05	0·05	0·07	0·04	0·05	0·06	0·04
North Africa and Middle East	Female	0·04	0·05	0·03	0·03	0·04	0·02	0·04	0·05	0·03	0·04	0·05	0·03
Oceania	Female	0·04	0·06	0·03	0·03	0·05	0·02	0·04	0·06	0·03	0·03	0·05	0·02
South Asia	Female	0·05	0·07	0·03	0·04	0·06	0·03	0·05	0·07	0·04	0·04	0·06	0·03
Southeast Asia	Female	0·05	0·07	0·04	0·04	0·06	0·03	0·06	0·08	0·05	0·05	0·07	0·04
Southern Latin America	Female	0·07	0·09	0·05	0·06	0·08	0·05	0·07	0·09	0·06	0·07	0·08	0·05
Southern sub-Saharan Africa	Female	0·06	0·08	0·05	0·05	0·07	0·04	0·06	0·07	0·05	0·05	0·06	0·04
Tropical Latin America	Female	0·05	0·07	0·04	0·04	0·06	0·03	0·05	0·07	0·04	0·05	0·06	0·04
Western Europe	Female	0·07	0·09	0·06	0·06	0·08	0·05	0·06	0·08	0·05	0·06	0·07	0·05
Western sub-Saharan Africa	Female	0·04	0·06	0·03	0·03	0·04	0·02	0·04	0·05	0·03	0·03	0·04	0·02
global	Male	0·06	0·10	0·05	0·06	0·09	0·04	0·06	0·09	0·05	0·06	0·08	0·04
High-middle SDI	Male	0·07	0·10	0·04	0·06	0·10	0·04	0·06	0·09	0·05	0·06	0·09	0·04
High SDI	Male	0·06	0·08	0·05	0·06	0·08	0·04	0·06	0·07	0·05	0·06	0·07	0·05
Low-middle SDI	Male	0·06	0·09	0·04	0·05	0·08	0·03	0·06	0·08	0·04	0·05	0·08	0·04
Low SDI	Male	0·05	0·08	0·04	0·04	0·06	0·03	0·05	0·07	0·04	0·04	0·06	0·03
Middle SDI	Male	0·07	0·12	0·04	0·06	0·10	0·04	0·06	0·09	0·04	0·06	0·09	0·04
Andean Latin America	Male	0·05	0·11	0·03	0·04	0·09	0·02	0·05	0·10	0·03	0·05	0·09	0·03
Australasia	Male	0·07	0·08	0·05	0·07	0·08	0·05	0·06	0·07	0·05	0·06	0·07	0·05
Caribbean	Male	0·05	0·07	0·03	0·04	0·06	0·03	0·05	0·06	0·03	0·05	0·06	0·03
Central Asia	Male	0·08	0·13	0·05	0·07	0·12	0·05	0·06	0·09	0·04	0·05	0·08	0·03
Central Europe	Male	0·06	0·09	0·04	0·06	0·08	0·04	0·07	0·08	0·05	0·06	0·08	0·05
Central Latin America	Male	0·04	0·09	0·03	0·04	0·07	0·02	0·05	0·08	0·03	0·05	0·08	0·03
Central sub-Saharan Africa	Male	0·06	0·09	0·04	0·05	0·08	0·03	0·06	0·08	0·04	0·06	0·08	0·04
East Asia	Male	0·08	0·13	0·05	0·07	0·11	0·04	0·07	0·11	0·04	0·07	0·11	0·04
Eastern Europe	Male	0·07	0·11	0·05	0·07	0·11	0·04	0·07	0·09	0·05	0·06	0·09	0·04
Eastern Sub-Saharan Africa	Male	0·06	0·08	0·04	0·04	0·06	0·03	0·06	0·08	0·04	0·05	0·06	0·03
High-income Asia Pacific	Male	0·07	0·13	0·04	0·07	0·13	0·04	0·07	0·10	0·05	0·07	0·10	0·05
High-income North America	Male	0·06	0·07	0·04	0·06	0·07	0·04	0·05	0·06	0·04	0·05	0·06	0·04
North Africa and Middle East	Male	0·04	0·06	0·03	0·03	0·05	0·02	0·04	0·06	0·03	0·04	0·05	0·03
Oceania	Male	0·06	0·09	0·04	0·05	0·08	0·03	0·06	0·09	0·04	0·05	0·08	0·04
South Asia	Male	0·05	0·08	0·04	0·05	0·07	0·03	0·06	0·07	0·04	0·05	0·07	0·04
Southeast Asia	Male	0·07	0·09	0·05	0·06	0·08	0·04	0·07	0·09	0·06	0·07	0·09	0·06
Southern Latin America	Male	0·07	0·10	0·05	0·07	0·09	0·05	0·07	0·09	0·05	0·07	0·09	0·05
Southern sub-Saharan Africa	Male	0·08	0·10	0·06	0·08	0·10	0·06	0·07	0·09	0·06	0·07	0·09	0·06
Tropical Latin America	Male	0·05	0·09	0·03	0·05	0·08	0·03	0·05	0·07	0·04	0·05	0·07	0·04
Western Europe	Male	0·06	0·08	0·04	0·06	0·07	0·04	0·06	0·07	0·05	0·06	0·07	0·04
Western sub-Saharan Africa	Male	0·05	0·07	0·03	0·04	0·06	0·03	0·05	0·07	0·04	0·04	0·06	0·03

From 1990 to 2019, Western Sub Saharan Africa, Oceania, and Southeastern Asia experienced an increase in ASDR and EAPC due to dietary risk factors. The largest rise in ASDR EAPC was observed in East Asia, Tropical Latin America, Southeastern Asia, Andean Latin America, Central Latin America for meat consumption, and processed meat. Central Sub-Saharan Africa showed low fiber intake, while High-Income North America had low vegetable intake, and Western Sub-Saharan Africa had low fruit intake. The highest increase in annual standardized DALY rates for EAPC cancer was linked to high processed meat consumption in East Asia, South Asia, Tropical Latin America, and Andean Latin America, high red meat intake in Southeast Asia, and low fiber intake in Sub-Saharan Africa. Oceania had low polyunsaturated fatty acid intake, Eastern Europe had low milk intake, and Central Latin America had low grain intake. Additionally, High-Income North America had low vegetable intake, and Central Sub-Saharan Africa had low fruit and fiber intake (Fig. 3).

### Relationship between SDI values with dietary risk factors

**Figure 4.**
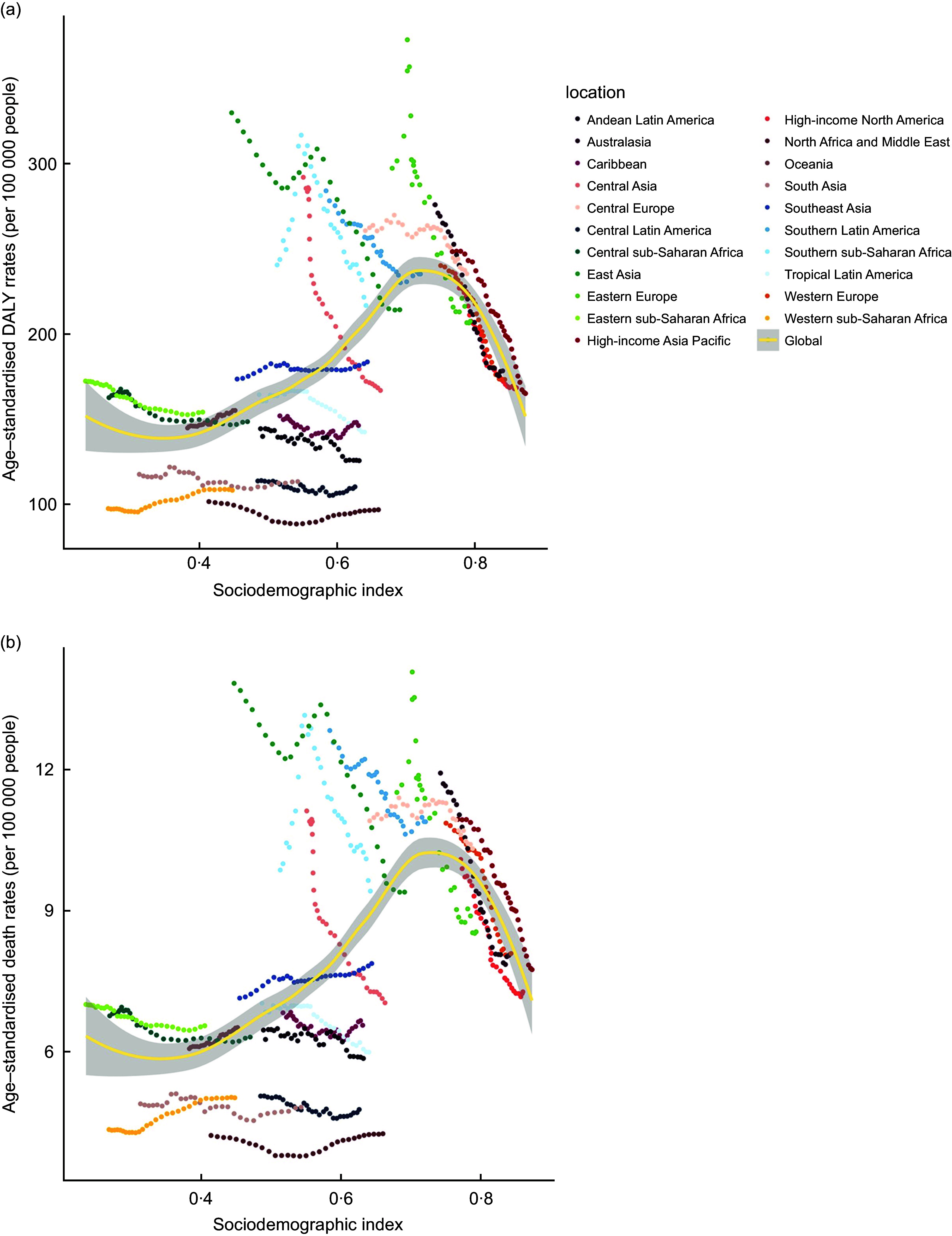
Age-standardized rates attributable to dietary factors across 21 Global Burden of Disease regions by sociodemographic index, 1990–2019. (a) DALYs. (b) Death. DALY, disability-adjusted life year.

**Figure 5.**
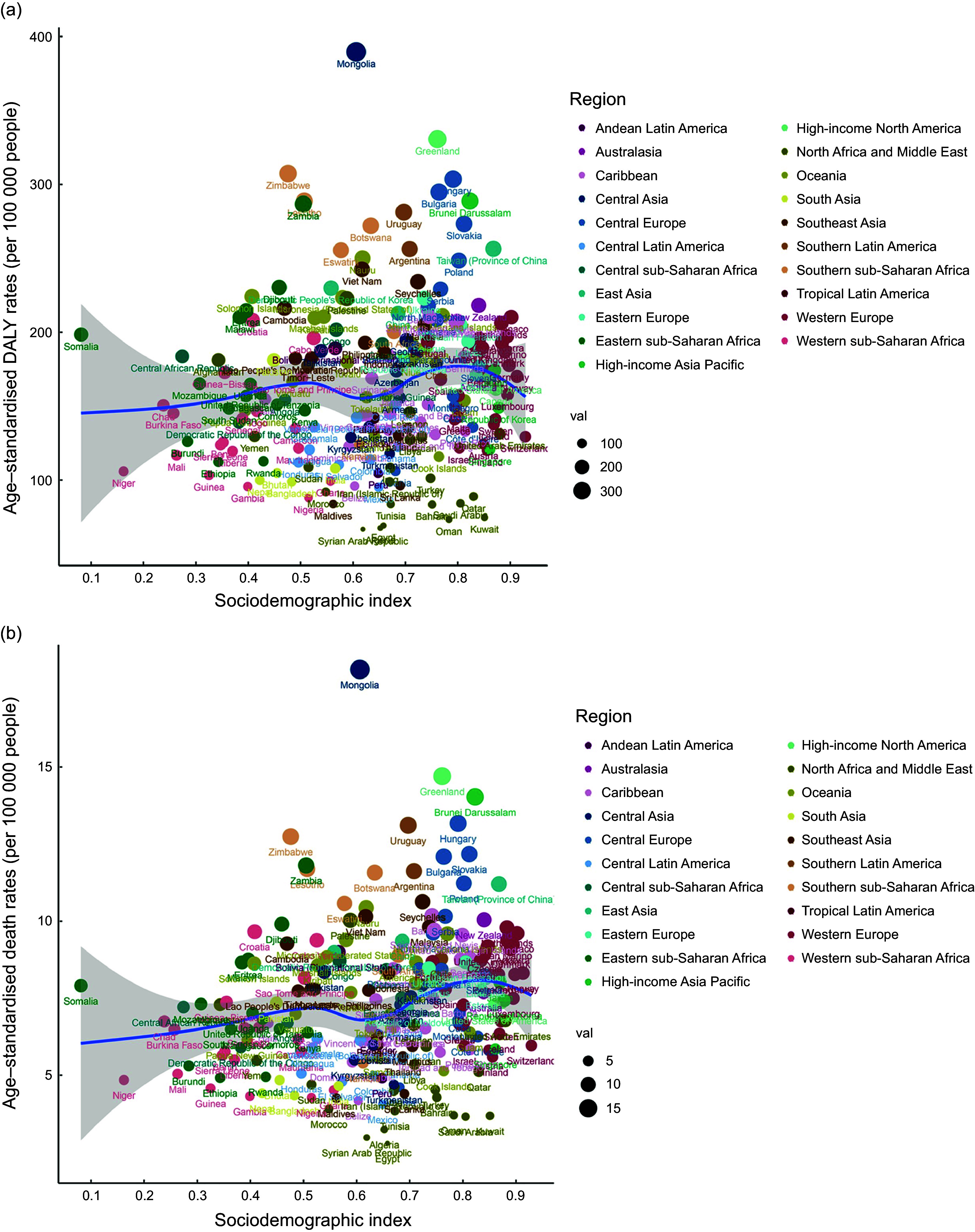
Age-standardized rates attributable to dietary factors across 204 countries and territories by sociodemographic index, 1990–2019. (a) DALYs. (b) Death. DALY, disability-adjusted life year.

As the SDI value increases, the cancer ASDR and DALY rates caused by dietary risk factors generally decrease across most regions. However, there is a slight upward trend in areas with lower SDI values, while a notable decline is observed in regions with higher SDI values. East Asia and Eastern Europe exhibit significant peaks in ASDR and DALY rates caused by dietary risk factors. In 1990, East Asia had an SDI close to 0·7, with an ASDR of 13·83 and a DALY rate of 329·49 per 100 000 people for cancer caused by dietary risk factors, significantly higher than other regions. By 2019, despite improvements, East Asia still had higher cancer ASDR (9·40/100 000) and DALY rates (213·93/100 000) compared to most regions. Moreover, Eastern Europe experiences a much higher cancer burden compared to regions with similar SDI values. The trend depicted in Fig. 5 illustrates that from 1990 to 2019, among 204 countries, cancer DALY and mortality rates attributable to dietary risk factors initially increased and then decreased with rising SDI values, peaking at 0·6. In 2019, Mongolia had the highest cancer ASDR (18·16/100 000) and DALY rates (389·57/100 000) attributable to dietary risk factors per 100 000 people. In contrast, Egypt had the lowest ASDR (2·79/100 000), and the Syrian Arab Republic had the lowest ASDR (2·79/100 000), and the lowest age-standardized DALY rate (66·73). (Figs 4 and 5)

## Discussion

This study thoroughly investigated the impact of dietary factors on cancer mortality and DALY, along with the regional specificity of each factor’s impact. The findings revealed a decreasing trend in cancer burden attributable to dietary factors from 1990 to 2019, while the number of deaths and disability-adjusted life years increased due to population growth and longer human lifespan. Key groups affected were the elderly aged 65 and men. The top three dietary risk factors contributing to the cancer burden were diets low in whole grains, milk, and calcium, predominantly found in the High middle SDI region, particularly in Southern Latin America, Central Europe, and Western Sub-Saharan Africa. Effective dietary pattern optimization in these regions necessitates a concerted effort led by local governments, in partnership with international health agencies such as the World Health Organization. Policy makers must prioritize comprehensive nutritional guidelines and enforce regulations that curb the availability of unhealthy food options. Empirical evidence from studies like He, Jenner, and MacGregor^(21)^ on the impact of modest salt reduction on blood pressure underscoreds the beneficial role of engaging community leaders to promote healthier eating habits and enhance local dietary regulations.

In 2019, the age-standardized death and DALY rates for cancer were approximately 5·5 %. This could be largely attributed to dietary factors, highlighting the importance of promoting a healthy diet to alleviate the burden of cancer. We identified three key contributors to cancer mortality and DALY rates: low intake of whole grains, low intake of milk, and cancer-related physiological and biological factors. Numerous meta-analysis studies have shown that higher consumption of whole grains is associated with a reduced risk of cancer in various specific areas such as the colon, stomach, pancreas, breast, esophagus, brain, and oral cavity. Additionally, dose-response analyses have indicated that a daily intake of 30 grams of whole grains could decrease the risk of cancer death by 7 %^(22)^. Notably, regions with higher SDI values tend to have lower whole grain intake and higher cancer mortality and DALY rates^(23)^. The low intake of whole grains is particularly prominent in Central Europe and Southern Latin America, where it is a significant risk factor for cancer^(24)^. Increased health awareness has sometimes led to the adoption of low-carb or gluten-free diets, which may inadvertently reduce whole grain intake, despite dietary guidelines recommending whole grains. Urbanization, nutritional transitions, changing dietary habits, and increasing health awareness are some of the factors contributing to the lower consumption of whole grains in these regions^(25)^. Interestingly, as SDI values increase, the burden of cancer stabilizes, but then decreases as SDI values approach 0·78 before rising again. Southern Latin America exhibited a turning point in cancer burden when the SDI value reached 0·6, after which it began to decline. Overall, the low intake of whole grains may be linked to various factors such as urbanization, dietary transitions, changing habits, and increased health consciousness^(26,27)^.

The presence of vitamin D, calcium, lactoferrin, conjugated linoleic acid, butyric acid, and lactose in milk provides certain anti-cancer properties^(28)^. Inadequate intake of vitamin D and calcium is a significant risk factor for various cancers^(29)^. Regions with low milk and calcium consumption exhibit higher cancer mortality rates, with Southern Latin America having the lowest milk intake and Southern Asia having the lowest calcium intake. As the SDI increases, the burden trend in Southeast Asia remains relatively stable. It is recommended that healthy adults under the age of 60 consume 1000 milligrams of calcium per day, with higher values for the elderly^(29)^. Evidence suggests that calcium deficiency is not only common in the elderly but also in young individuals^(30)^, particularly in Southern Latin America and Southeast Asia where low calcium intake is prevalent^(31)^. Addressing the issue of low calcium intake is crucial for improving nutritional quality and promoting cancer prevention through dietary control.

Through visual comparison of bar charts, it could be observed that the age-standardized mortality rate and DALY rate of cancer attributed to dietary risk factors are higher in males than in females. Additionally, the age at which cancer deaths peak is higher in females compared to males. The influence of diet on cancer risk may be linked to the protective impact of estrogen^(32)^. Following a comprehensive analysis of dietary factors, significant sex disparities persist. Dietary habits, particularly in males, show higher intake of red and processed meat, while females exhibit higher consumption of milk, dairy products, and calcium due to various complex interactions^(33)^. While low calcium intake may contribute to higher cancer rates in older individuals^(30)^, it is not likely to be the only factor. Other age-related biological changes and cumulative risk factors must also be considered. Therefore, providing effective and appropriate dietary guidance for the elderly is crucial, as they may find it more challenging to make dietary changes.

Regions with high SDI values tend to have higher consumption of red and processed meat, leading to an increased burden of cancer. Red meat and processed meat are favored for their sensory qualities and cultural significance, with meat consumption on the rise globally^(34)^. However, emerging research indicates that red meat could generate carcinogens like N-nitrosamines, polycyclic aromatic hydrocarbons, or nitrites during various cooking or processing methods^(35,36)^. Compared to populations with the lowest red and processed meat intake, those with the highest consumption experienced a 23 % and 29 % increase in all-cause mortality, respectively^(37)^. Processed meat, in particular, contains high levels of sodium and nitrites^(38)^, reducing its intake could help manage sodium consumption. In regions with medium SDI region, the cancer burden linked to dietary risks is primarily due to excessive sodium intake. Despite a notable reduction in cancer burden associated with high sodium intake across regions from 1990 to 2019, East Asia and Andean Latin America continue to have elevated salt consumption, highlighting the urgent need for shifts in health concepts and dietary habits. Conversely, in low and medium-low SDI regions, the cancer burden from dietary risks is mainly attributed to low fruit and vegetable intake. The cancer-fighting potential of consuming ample fresh fruits and vegetables is well-documented due to their fiber, vitamins, minerals, and antioxidants^(39)^. The availability and affordability of fruits and vegetables play a crucial role in determining consumption levels, necessitating support from national policies, regulations, taxation, and regional coordination^(40,41)^.

When comparing cancer mortality across regions, it’s important to account for not only cancer risk factors but also other competing causes of death and their associated risks. Mongolia bears the highest cancer burden, while Egypt and the Syrian Arab Republic have the lowest burden. This disparity could be attributed to the dietary practices prevalent in these countries. Mongolia’s nomadic diet is characterized by high meat consumption, inadequate food preservation and refrigeration facilities, potential excessive salt intake, low consumption of fruits and vegetables, and frequent alcohol consumption, all of which are potential cancer risk factors^(42)^. Egypt’s notably low cancer rates may be linked to a diet rich in high-fiber foods, beans, and green vegetables^(43,44)^. Their consumption of protein and fat falls below Western standards, with a majority of protein derived from plant sources and a significant portion of the diet comprising fiber^(45)^. While the identification and adoption of healthier dietary patterns are integral to overall healthy lifestyle choices, they may partially explain the reduced cancer risk observed in these regions. Dietary habits are deeply intertwined with cultural practices and specific factors unique to each country^(46)^. Governments, in collaboration with public health organizations and community leaders, are responsible for guiding these changes through the implementation of health promotion campaigns, nutritional education programs, and policy regulations tailored to cultural contexts.

If a country struggles to change its dietary patterns, it is crucial to prioritize population-wide dietary adjustments to lessen the burden of cancer. Effective policy interventions that have been successful in other contexts include Norway’s taxation on sugar-sweetened beverages, Japan’s national dietary guidelines which emphasize the consumption of *n*-3 fatty acids and seafood^(47)^, and Brazil’s government-led campaigns to promote fruit and vegetable consumption^(48)^. This study offers a detailed analysis of cancer mortality and DALY rates attributed to dietary factors between 1990 and 2019 using GBD data to highlight regional disparities in cancer burden from dietary factors. However, the study faces several limitations that suggest areas for further research. Firstly, the potential interactions between dietary risk factors might lead to underestimation or overestimation of their impacts. Future studies should explore the independence and interdependence of these factors using more localized datasets. Secondly, the limitations in the modeling methods of the GBD2019 database highlight the need for developing more refined models that could accurately reflect the nuances of dietary impacts on different cancer types. Thirdly, although adjustments were made for confounding factors like age and sex, future research should consider the effects of other potential confounders such as socioeconomic status and genetic predispositions, particularly in longitudinal cohort studies.

The cancer burden attributable to dietary factors exhibited a declining trend from 1990 to 2019. Globally, inadequate consumption of whole grains, milk, and calcium emerged as the primary risk factors for cancer. Regionally, the High SDI region has been reducing red and processed meat consumption, the Middle SDI region is focusing on limiting sodium intake, and the Low SDI region is emphasizing increased consumption of vegetables and fruits. Specific recommendations include increasing whole grains intake in Central Europe, boosting milk intake in Southern Latin America, and enhancing calcium intake in Southeast Asia. Urgent attention is needed in the Southern Latin America region and Mongolia to establish healthier dietary patterns. Targeting elderly and male populations is crucial in addressing specific dietary needs. A thorough evaluation of the impact of various dietary risk factors on cancer burden can offer valuable insights to inform individual dietary choices, cancer prevention strategies, and health policy development.

## Supporting information

10.1017/S1368980024002489.sm001 Xie and Zhao supplementary material Xie and Zhao supplementary material

## Supplementary material

For supplementary material accompanying this paper visit https://doi.org/10.1017/S1368980024002489
