# Supplementary material for: The global burden of cancer attributable to dietary factors from 1990 to 2019
Source: Public Health Nutr. 2025 Jan 9;29(1):e106. doi: 10.1017/S1368980024002489 (PMC13434562; doi:10.1017/S1368980024002489)
Supplement: Supplementary file 1 [file S1368980024002489sup001.doc]

**Table S1**. Cancer deaths and DALYs (Disability-Adjusted Life Years) attributed to dietary risk factors and their 95% uncertainty intervals.

|  | **1990** | **1990** | **2019** | **2019** |
| --- | --- | --- | --- | --- |
| **Location** | **Deaths** | **DALYs (Disability-Adjusted Life Years)** | **Deaths** | **DALYs (Disability-Adjusted Life Years)** |
| global | 372355.76(269685.75,545341.00) | 9368997.08(6792059.60,13895472.55) | 605427.05(454445.44,811175.56) | 13951293.26(10502647.25,18791392.03) |
| **SDI** | - | - | - | - |
| High-middle SDI | 115373.13(81345.13,176283.99) | 2922062.20(2050380.15,4489132.02) | 168626.29(123242.94,229301.75) | 3817811.76(2775434.22,5204933.57) |
| High SDI | 113991.85(86572.61,148697.52) | 2512633.69(1898656.83,3294153.84) | 156364.89(118574.24,194062.23) | 3032456.74(2337515.96,3704816.89) |
| Low-middle SDI | 33456.19(23662.28,52881.26) | 944482.40(662245.35,1504153.43) | 76636.12(57067.47,108496.28) | 1991292.19(1479865.68,2845592.17) |
| Low SDI | 11983.27(8657.61,17980.48) | 338535.56(244841.96,509742.95) | 26009.00(19574.11,35543.28) | 712317.46(533134.61,977425.87) |
| Middle SDI | 97399.96(63090.57,155491.81) | 2647674.10(1729068.80,4245638.76) | 177501.42(126746.36,255329.69) | 4390757.83(3159405.44,6298039.84) |
| **Regions** | - | - | - | - |
| Andean Latin America | 1253.41(692.89,2685.00) | 31037.47(17145.61,66639.87) | 3205.75(1927.43,5947.39) | 71320.43(42663.25,132904.75) |
| Australasia | 2763.47(2180.19,3279.02) | 63513.96(50473.52,74875.41) | 4105.18(3184.82,4925.90) | 82545.53(64773.04,98069.28) |
| Caribbean | 1718.62(1291.33,2339.76) | 40134.65(30285.54,55154.41) | 3397.23(2449.17,4420.25) | 75436.89(54682.14,99518.19) |
| Central Asia | 5178.80(3450.36,8096.24) | 144840.24(97224.98,227591.00) | 4845.31(3378.18,7364.90) | 132627.80(91530.90,202788.13) |
| Central Europe | 15749.71(11122.25,21937.33) | 384083.30(271603.86,532683.50) | 22300.05(15908.52,28561.20) | 475356.94(337870.49,611117.17) |
| Central Latin America | 3981.75(2495.62,7492.60) | 101520.81(63844.43,189418.94) | 11068.61(7461.04,17333.66) | 265506.44(178366.02,417084.10) |
| Central sub-Saharan Africa | 1420.17(962.90,1999.78) | 40852.62(27545.62,57881.25) | 3101.06(2087.66,4209.59) | 88243.28(59281.28,119921.69) |
| East Asia | 114893.43(69013.24,192111.07) | 3093182.41(1876198.09,5171073.69) | 187008.21(120783.12,290349.71) | 4495533.06(2906208.36,6915897.58) |
| Eastern Europe | 32226.18(22864.05,49487.32) | 838739.34(586637.89,1292762.32) | 29522.29(21451.09,39804.68) | 689774.66(499904.18,933965.56) |
| Eastern Sub-Saharan Africa | 5011.14(3433.34,7038.99) | 142690.81(97246.83,202396.77) | 10009.77(7234.75,13249.68) | 278813.85(200765.02,370963.73) |
| High-income Asia Pacific | 21267.15(13263.40,37391.81) | 505444.54(314179.41,900139.54) | 38388.39(26525.02,55814.31) | 668699.89(482164.97,953086.57) |
| High-income North America | 35785.49(27674.04,43009.48) | 792848.05(615904.32,949048.01) | 46267.84(35843.18,55436.70) | 982530.05(765073.43,1173014.92) |
| North Africa and Middle East | 6840.02(4998.71,10604.57) | 190213.42(135477.72,295351.56) | 17222.72(12561.41,23125.98) | 452879.40(331687.82,616606.27) |
| Oceania | 164.39(113.71,253.16) | 4845.71(3390.22,7394.02) | 421.52(288.10,641.99) | 12459.00(8526.46,19046.90) |
| South Asia | 25677.46(18490.32,38911.66) | 732734.27(522810.57,1132116.47) | 64181.01(47732.40,88243.27) | 1671009.41(1221018.38,2330707.09) |
| Southeast Asia | 17385.60(13008.41,24249.06) | 486940.95(365078.43,681253.49) | 45278.20(34596.61,57441.55) | 1182012.01(906866.27,1494462.92) |
| Southern Latin America | 5722.73(4339.68,7720.91) | 131517.45(99889.00,176772.87) | 9180.57(7002.20,11614.18) | 191903.72(147181.59,243192.87) |
| Southern sub-Saharan Africa | 2613.59(1942.68,3431.27) | 71683.03(53482.08,93863.18) | 4995.95(3875.58,6089.10) | 129361.14(99279.28,158537.29) |
| Tropical Latin America | 6042.07(4104.38,9759.23) | 160801.71(109951.04,255934.69) | 14324.62(10477.80,19709.70) | 352202.87(261523.03,477452.13) |
| Western Europe | 63182.61(48095.35,80113.47) | 1322019.48(1022313.97,1669966.94) | 78233.88(59472.05,95522.31) | 1437968.34(1104825.31,1742521.65) |
| Western sub-Saharan Africa | 3477.94(2528.69,5403.58) | 89352.86(64569.92,139370.15) | 8368.89(6320.25,11737.14) | 215108.56(160404.63,299262.18) |

**Table 2.** In 21 regions, cancer death/disability caused by dietary risk factors resulted in the total PAF value of adjusted life year (; 95%UI：）

|  |  | **1990** | **1990** | **1990** | **1990** | **1990** | **1990** | **2019** | **2019** | **2019** | **2019** | **2019** | **2019** |
| --- | --- | --- | --- | --- | --- | --- | --- | --- | --- | --- | --- | --- | --- |
|  | **sex** | **Deaths** | **Deaths** | **Deaths** | **DALYs** | **DALYs** | **DALYs** | **Deaths** | **Deaths** | **Deaths** | **DALYs** | **DALYs** | **DALYs** |
|  |  | val | upper | lower | val | upper | lower | val | upper | lower | val | upper | lower |
| global | both | 0.06 | 0.09 | 0.05 | 0.06 | 0.08 | 0.04 | 0.06 | 0.08 | 0.05 | 0.06 | 0.08 | 0.04 |
| High-middle SDI | both | 0.07 | 0.10 | 0.05 | 0.06 | 0.09 | 0.04 | 0.06 | 0.09 | 0.05 | 0.06 | 0.08 | 0.04 |
| High SDI | both | 0.07 | 0.09 | 0.05 | 0.06 | 0.08 | 0.05 | 0.06 | 0.07 | 0.05 | 0.06 | 0.07 | 0.05 |
| Low-middle SDI | both | 0.05 | 0.08 | 0.04 | 0.04 | 0.07 | 0.03 | 0.06 | 0.08 | 0.04 | 0.05 | 0.07 | 0.04 |
| Low SDI | both | 0.05 | 0.07 | 0.03 | 0.04 | 0.06 | 0.03 | 0.05 | 0.07 | 0.04 | 0.04 | 0.05 | 0.03 |
| Middle SDI | both | 0.07 | 0.11 | 0.04 | 0.06 | 0.09 | 0.04 | 0.06 | 0.09 | 0.04 | 0.06 | 0.08 | 0.04 |
| Andean Latin America | both | 0.05 | 0.10 | 0.03 | 0.04 | 0.08 | 0.02 | 0.05 | 0.09 | 0.03 | 0.04 | 0.08 | 0.03 |
| Australasia | both | 0.07 | 0.09 | 0.06 | 0.07 | 0.08 | 0.06 | 0.06 | 0.08 | 0.05 | 0.06 | 0.07 | 0.05 |
| Caribbean | both | 0.05 | 0.07 | 0.04 | 0.04 | 0.06 | 0.03 | 0.05 | 0.06 | 0.04 | 0.04 | 0.06 | 0.03 |
| Central Asia | both | 0.08 | 0.12 | 0.05 | 0.07 | 0.10 | 0.05 | 0.05 | 0.08 | 0.04 | 0.05 | 0.08 | 0.04 |
| Central Europe | both | 0.06 | 0.09 | 0.04 | 0.06 | 0.08 | 0.04 | 0.06 | 0.08 | 0.05 | 0.06 | 0.07 | 0.05 |
| Central Latin America | both | 0.04 | 0.08 | 0.03 | 0.03 | 0.06 | 0.02 | 0.05 | 0.07 | 0.03 | 0.04 | 0.07 | 0.03 |
| Central sub-Saharan Africa | both | 0.05 | 0.07 | 0.04 | 0.04 | 0.06 | 0.03 | 0.05 | 0.07 | 0.04 | 0.05 | 0.06 | 0.03 |
| East Asia | both | 0.08 | 0.13 | 0.05 | 0.07 | 0.11 | 0.04 | 0.07 | 0.10 | 0.04 | 0.06 | 0.10 | 0.04 |
| Eastern Europe | both | 0.07 | 0.11 | 0.05 | 0.07 | 0.11 | 0.05 | 0.07 | 0.09 | 0.05 | 0.06 | 0.08 | 0.05 |
| Eastern Sub-Saharan Africa | both | 0.05 | 0.07 | 0.04 | 0.04 | 0.06 | 0.02 | 0.05 | 0.07 | 0.04 | 0.04 | 0.05 | 0.03 |
| High-income Asia Pacific | both | 0.07 | 0.13 | 0.05 | 0.07 | 0.12 | 0.04 | 0.07 | 0.10 | 0.05 | 0.07 | 0.10 | 0.05 |
| High-income North America | both | 0.06 | 0.07 | 0.05 | 0.06 | 0.07 | 0.04 | 0.05 | 0.06 | 0.04 | 0.05 | 0.06 | 0.04 |
| North Africa and Middle East | both | 0.04 | 0.06 | 0.03 | 0.03 | 0.05 | 0.02 | 0.04 | 0.05 | 0.03 | 0.04 | 0.05 | 0.03 |
| Oceania | both | 0.05 | 0.07 | 0.03 | 0.04 | 0.06 | 0.03 | 0.05 | 0.07 | 0.04 | 0.04 | 0.06 | 0.03 |
| South Asia | both | 0.05 | 0.08 | 0.04 | 0.04 | 0.06 | 0.03 | 0.05 | 0.07 | 0.04 | 0.04 | 0.06 | 0.03 |
| Southeast Asia | both | 0.06 | 0.08 | 0.05 | 0.05 | 0.07 | 0.04 | 0.07 | 0.08 | 0.06 | 0.06 | 0.08 | 0.05 |
| Southern Latin America | both | 0.07 | 0.09 | 0.05 | 0.06 | 0.08 | 0.05 | 0.07 | 0.09 | 0.06 | 0.07 | 0.08 | 0.05 |
| Southern sub-Saharan Africa | both | 0.07 | 0.09 | 0.05 | 0.07 | 0.08 | 0.05 | 0.07 | 0.08 | 0.05 | 0.06 | 0.08 | 0.05 |
| Tropical Latin America | both | 0.05 | 0.08 | 0.03 | 0.04 | 0.07 | 0.03 | 0.05 | 0.07 | 0.04 | 0.05 | 0.07 | 0.04 |
| Western Europe | both | 0.06 | 0.08 | 0.05 | 0.06 | 0.08 | 0.05 | 0.06 | 0.07 | 0.05 | 0.06 | 0.07 | 0.05 |
| Western sub-Saharan Africa | both | 0.04 | 0.07 | 0.03 | 0.03 | 0.05 | 0.03 | 0.04 | 0.06 | 0.03 | 0.04 | 0.05 | 0.03 |
| global | female | 0.06 | 0.09 | 0.05 | 0.05 | 0.08 | 0.04 | 0.06 | 0.08 | 0.04 | 0.05 | 0.07 | 0.04 |
| High-middle SDI | female | 0.07 | 0.10 | 0.05 | 0.06 | 0.09 | 0.04 | 0.06 | 0.08 | 0.05 | 0.06 | 0.07 | 0.04 |
| High SDI | female | 0.07 | 0.09 | 0.05 | 0.06 | 0.08 | 0.05 | 0.06 | 0.07 | 0.05 | 0.06 | 0.07 | 0.05 |
| Low-middle SDI | female | 0.05 | 0.08 | 0.04 | 0.04 | 0.06 | 0.03 | 0.05 | 0.07 | 0.04 | 0.04 | 0.06 | 0.03 |
| Low SDI | female | 0.04 | 0.06 | 0.03 | 0.03 | 0.05 | 0.02 | 0.04 | 0.06 | 0.03 | 0.03 | 0.05 | 0.03 |
| Middle SDI | female | 0.06 | 0.10 | 0.04 | 0.05 | 0.08 | 0.04 | 0.06 | 0.08 | 0.04 | 0.05 | 0.07 | 0.04 |
| Andean Latin America | female | 0.04 | 0.09 | 0.03 | 0.03 | 0.07 | 0.02 | 0.05 | 0.08 | 0.03 | 0.04 | 0.07 | 0.03 |
| Australasia | female | 0.08 | 0.09 | 0.06 | 0.07 | 0.09 | 0.06 | 0.07 | 0.08 | 0.05 | 0.07 | 0.08 | 0.05 |
| Caribbean | female | 0.05 | 0.06 | 0.04 | 0.04 | 0.05 | 0.03 | 0.05 | 0.06 | 0.04 | 0.04 | 0.05 | 0.03 |
| Central Asia | female | 0.07 | 0.11 | 0.05 | 0.06 | 0.09 | 0.05 | 0.05 | 0.07 | 0.04 | 0.05 | 0.07 | 0.03 |
| Central Europe | female | 0.06 | 0.08 | 0.04 | 0.05 | 0.07 | 0.04 | 0.06 | 0.08 | 0.05 | 0.06 | 0.07 | 0.04 |
| Central Latin America | female | 0.04 | 0.07 | 0.03 | 0.03 | 0.05 | 0.02 | 0.05 | 0.07 | 0.03 | 0.04 | 0.06 | 0.03 |
| Central sub-Saharan Africa | female | 0.04 | 0.06 | 0.03 | 0.03 | 0.05 | 0.02 | 0.04 | 0.06 | 0.03 | 0.04 | 0.05 | 0.03 |
| East Asia | female | 0.07 | 0.12 | 0.05 | 0.06 | 0.10 | 0.04 | 0.06 | 0.09 | 0.04 | 0.06 | 0.09 | 0.04 |
| Eastern Europe | female | 0.08 | 0.11 | 0.06 | 0.07 | 0.10 | 0.05 | 0.07 | 0.09 | 0.05 | 0.06 | 0.08 | 0.04 |
| Eastern Sub-Saharan Africa | female | 0.05 | 0.07 | 0.03 | 0.03 | 0.05 | 0.02 | 0.05 | 0.06 | 0.04 | 0.04 | 0.05 | 0.03 |
| High-income Asia Pacific | female | 0.07 | 0.12 | 0.05 | 0.07 | 0.11 | 0.04 | 0.07 | 0.10 | 0.05 | 0.07 | 0.09 | 0.05 |
| High-income North America | female | 0.06 | 0.08 | 0.05 | 0.06 | 0.07 | 0.05 | 0.05 | 0.07 | 0.04 | 0.05 | 0.06 | 0.04 |
| North Africa and Middle East | female | 0.04 | 0.05 | 0.03 | 0.03 | 0.04 | 0.02 | 0.04 | 0.05 | 0.03 | 0.04 | 0.05 | 0.03 |
| Oceania | female | 0.04 | 0.06 | 0.03 | 0.03 | 0.05 | 0.02 | 0.04 | 0.06 | 0.03 | 0.03 | 0.05 | 0.02 |
| South Asia | female | 0.05 | 0.07 | 0.03 | 0.04 | 0.06 | 0.03 | 0.05 | 0.07 | 0.04 | 0.04 | 0.06 | 0.03 |
| Southeast Asia | female | 0.05 | 0.07 | 0.04 | 0.04 | 0.06 | 0.03 | 0.06 | 0.08 | 0.05 | 0.05 | 0.07 | 0.04 |
| Southern Latin America | female | 0.07 | 0.09 | 0.05 | 0.06 | 0.08 | 0.05 | 0.07 | 0.09 | 0.06 | 0.07 | 0.08 | 0.05 |
| Southern sub-Saharan Africa | female | 0.06 | 0.08 | 0.05 | 0.05 | 0.07 | 0.04 | 0.06 | 0.07 | 0.05 | 0.05 | 0.06 | 0.04 |
| Tropical Latin America | female | 0.05 | 0.07 | 0.04 | 0.04 | 0.06 | 0.03 | 0.05 | 0.07 | 0.04 | 0.05 | 0.06 | 0.04 |
| Western Europe | female | 0.07 | 0.09 | 0.06 | 0.06 | 0.08 | 0.05 | 0.06 | 0.08 | 0.05 | 0.06 | 0.07 | 0.05 |
| Western sub-Saharan Africa | female | 0.04 | 0.06 | 0.03 | 0.03 | 0.04 | 0.02 | 0.04 | 0.05 | 0.03 | 0.03 | 0.04 | 0.02 |
| global | male | 0.06 | 0.10 | 0.05 | 0.06 | 0.09 | 0.04 | 0.06 | 0.09 | 0.05 | 0.06 | 0.08 | 0.04 |
| High-middle SDI | male | 0.07 | 0.10 | 0.04 | 0.06 | 0.10 | 0.04 | 0.06 | 0.09 | 0.05 | 0.06 | 0.09 | 0.04 |
| High SDI | male | 0.06 | 0.08 | 0.05 | 0.06 | 0.08 | 0.04 | 0.06 | 0.07 | 0.05 | 0.06 | 0.07 | 0.05 |
| Low-middle SDI | male | 0.06 | 0.09 | 0.04 | 0.05 | 0.08 | 0.03 | 0.06 | 0.08 | 0.04 | 0.05 | 0.08 | 0.04 |
| Low SDI | male | 0.05 | 0.08 | 0.04 | 0.04 | 0.06 | 0.03 | 0.05 | 0.07 | 0.04 | 0.04 | 0.06 | 0.03 |
| Middle SDI | male | 0.07 | 0.12 | 0.04 | 0.06 | 0.10 | 0.04 | 0.06 | 0.09 | 0.04 | 0.06 | 0.09 | 0.04 |
| Andean Latin America | male | 0.05 | 0.11 | 0.03 | 0.04 | 0.09 | 0.02 | 0.05 | 0.10 | 0.03 | 0.05 | 0.09 | 0.03 |
| Australasia | male | 0.07 | 0.08 | 0.05 | 0.07 | 0.08 | 0.05 | 0.06 | 0.07 | 0.05 | 0.06 | 0.07 | 0.05 |
| Caribbean | male | 0.05 | 0.07 | 0.03 | 0.04 | 0.06 | 0.03 | 0.05 | 0.06 | 0.03 | 0.05 | 0.06 | 0.03 |
| Central Asia | male | 0.08 | 0.13 | 0.05 | 0.07 | 0.12 | 0.05 | 0.06 | 0.09 | 0.04 | 0.05 | 0.08 | 0.03 |
| Central Europe | male | 0.06 | 0.09 | 0.04 | 0.06 | 0.08 | 0.04 | 0.07 | 0.08 | 0.05 | 0.06 | 0.08 | 0.05 |
| Central Latin America | male | 0.04 | 0.09 | 0.03 | 0.04 | 0.07 | 0.02 | 0.05 | 0.08 | 0.03 | 0.05 | 0.08 | 0.03 |
| Central sub-Saharan Africa | male | 0.06 | 0.09 | 0.04 | 0.05 | 0.08 | 0.03 | 0.06 | 0.08 | 0.04 | 0.06 | 0.08 | 0.04 |
| East Asia | male | 0.08 | 0.13 | 0.05 | 0.07 | 0.11 | 0.04 | 0.07 | 0.11 | 0.04 | 0.07 | 0.11 | 0.04 |
| Eastern Europe | male | 0.07 | 0.11 | 0.05 | 0.07 | 0.11 | 0.04 | 0.07 | 0.09 | 0.05 | 0.06 | 0.09 | 0.04 |
| Eastern Sub-Saharan Africa | male | 0.06 | 0.08 | 0.04 | 0.04 | 0.06 | 0.03 | 0.06 | 0.08 | 0.04 | 0.05 | 0.06 | 0.03 |
| High-income Asia Pacific | male | 0.07 | 0.13 | 0.04 | 0.07 | 0.13 | 0.04 | 0.07 | 0.10 | 0.05 | 0.07 | 0.10 | 0.05 |
| High-income North America | male | 0.06 | 0.07 | 0.04 | 0.06 | 0.07 | 0.04 | 0.05 | 0.06 | 0.04 | 0.05 | 0.06 | 0.04 |
| North Africa and Middle East | male | 0.04 | 0.06 | 0.03 | 0.03 | 0.05 | 0.02 | 0.04 | 0.06 | 0.03 | 0.04 | 0.05 | 0.03 |
| Oceania | male | 0.06 | 0.09 | 0.04 | 0.05 | 0.08 | 0.03 | 0.06 | 0.09 | 0.04 | 0.05 | 0.08 | 0.04 |
| South Asia | male | 0.05 | 0.08 | 0.04 | 0.05 | 0.07 | 0.03 | 0.06 | 0.07 | 0.04 | 0.05 | 0.07 | 0.04 |
| Southeast Asia | male | 0.07 | 0.09 | 0.05 | 0.06 | 0.08 | 0.04 | 0.07 | 0.09 | 0.06 | 0.07 | 0.09 | 0.06 |
| Southern Latin America | male | 0.07 | 0.10 | 0.05 | 0.07 | 0.09 | 0.05 | 0.07 | 0.09 | 0.05 | 0.07 | 0.09 | 0.05 |
| Southern sub-Saharan Africa | male | 0.08 | 0.10 | 0.06 | 0.08 | 0.10 | 0.06 | 0.07 | 0.09 | 0.06 | 0.07 | 0.09 | 0.06 |
| Tropical Latin America | male | 0.05 | 0.09 | 0.03 | 0.05 | 0.08 | 0.03 | 0.05 | 0.07 | 0.04 | 0.05 | 0.07 | 0.04 |
| Western Europe | male | 0.06 | 0.08 | 0.04 | 0.06 | 0.07 | 0.04 | 0.06 | 0.07 | 0.05 | 0.06 | 0.07 | 0.04 |
| Western sub-Saharan Africa | male | 0.05 | 0.07 | 0.03 | 0.04 | 0.06 | 0.03 | 0.05 | 0.07 | 0.04 | 0.04 | 0.06 | 0.03 |

**Table S3.** Age-standardized cancer mortality attributable to dietary risk factors in 204 countries in 2019 (; 95%UI:) Age standardized DALY rate (; 95% UI:) EAPC value (95%CI:)

|  | **2019** | **1990-2019** | **2019** | **1990-2019** |
| --- | --- | --- | --- | --- |
|  | **Deaths** | **EAPC** | **DALYs** | **EAPC** |
| Afghanistan | 7.29(4.84,11.92) | -0.11(-0.15,-0.06) | 181.18(114.08,308.15) | -0.26(-0.33,-0.19) |
| Albania | 4.61(2.81,7.72) | -1.00(-1.23,-0.77) | 106.08(63.26,177.39) | -1.04(-1.28,-0.80) |
| Algeria | 3.24(2.22,4.27) | -0.84(-0.90,-0.77) | 67.52(45.65,89.43) | -1.06(-1.13,-0.99) |
| American Samoa | 8.67(6.36,12.14) | 0.12(0.01,0.22) | 192.25(138.94,271.87) | 0.13(0.02,0.24) |
| Andorra | 9.23(6.25,12.24) | -0.89(-0.95,-0.82) | 197.36(132.92,265.49) | -0.89(-0.94,-0.84) |
| Angola | 6.70(4.86,9.09) | -1.08(-1.20,-0.96) | 154.18(108.28,212.55) | -1.35(-1.49,-1.22) |
| Antigua and Barbuda | 7.51(5.41,10.12) | 0.35(0.20,0.50) | 151.40(108.93,205.39) | 0.09(-0.06,0.23) |
| Argentina | 11.62(8.98,14.26) | -0.44(-0.54,-0.34) | 256.24(199.96,312.04) | -0.50(-0.58,-0.42) |
| Armenia | 6.75(4.63,10.01) | -1.27(-1.37,-1.16) | 155.66(106.57,232.54) | -1.86(-1.99,-1.74) |
| Australia | 7.68(5.93,9.21) | -1.69(-1.81,-1.57) | 170.57(132.54,203.33) | -1.81(-1.94,-1.69) |
| Austria | 5.81(4.33,7.30) | -2.83(-2.96,-2.70) | 124.46(93.84,154.57) | -3.06(-3.22,-2.91) |
| Azerbaijan | 7.29(4.45,12.29) | -0.69(-0.87,-0.51) | 173.90(109.11,284.23) | -1.34(-1.55,-1.12) |
| Bahamas | 8.75(6.29,11.62) | 0.05(-0.03,0.13) | 206.80(147.08,276.28) | -0.05(-0.13,0.02) |
| Bahrain | 4.28(2.89,5.65) | -1.26(-1.52,-0.99) | 83.50(56.00,111.34) | -1.73(-1.97,-1.49) |
| Bangladesh | 4.34(2.81,6.58) | -0.91(-1.07,-0.76) | 98.91(62.69,150.55) | -1.13(-1.24,-1.01) |
| Barbados | 10.18(7.33,13.21) | 0.24(0.11,0.38) | 216.25(155.23,281.59) | 0.05(-0.04,0.14) |
| Belarus | 8.15(5.58,11.66) | -1.83(-2.13,-1.54) | 196.72(131.78,284.36) | -2.23(-2.54,-1.92) |
| Belgium | 8.25(6.24,10.05) | -1.52(-1.64,-1.39) | 174.01(132.18,210.51) | -1.52(-1.59,-1.46) |
| Belize | 4.18(2.80,6.49) | 0.57(0.13,1.00) | 95.98(63.93,149.11) | 0.77(0.33,1.20) |
| Benin | 5.77(3.96,9.00) | 0.40(0.32,0.48) | 125.57(84.34,196.27) | 0.23(0.17,0.30) |
| Bermuda | 8.82(6.47,11.35) | -0.88(-0.93,-0.83) | 183.26(134.15,236.54) | -0.97(-1.03,-0.91) |
| Bhutan | 4.84(3.11,7.16) | 0.22(0.15,0.29) | 104.70(64.17,157.09) | -0.22(-0.31,-0.14) |
| Bolivia (Plurinational State of) | 8.99(4.75,17.95) | -0.03(-0.10,0.04) | 189.16(101.91,378.95) | -0.31(-0.38,-0.23) |
| Bosnia and Herzegovina | 8.45(5.51,12.11) | 0.01(-0.15,0.16) | 189.45(122.46,276.96) | -0.32(-0.46,-0.18) |
| Botswana | 11.57(7.90,15.62) | -0.13(-0.49,0.22) | 272.05(177.19,377.81) | -0.30(-0.72,0.13) |
| Brazil | 5.99(4.39,8.25) | -0.60(-0.70,-0.50) | 142.18(105.71,192.82) | -0.53(-0.66,-0.41) |
| Brunei Darussalam | 14.03(10.09,18.10) | 0.01(-0.23,0.26) | 288.73(208.51,374.58) | -0.24(-0.53,0.06) |
| Bulgaria | 12.09(8.52,16.33) | 1.88(1.39,2.37) | 294.81(203.94,401.82) | 1.68(1.21,2.14) |
| Burkina Faso | 6.48(4.54,10.03) | 0.72(0.55,0.89) | 145.22(100.90,222.10) | 0.64(0.45,0.82) |
| Burundi | 5.30(3.24,8.12) | -1.28(-1.41,-1.16) | 126.14(75.48,194.72) | -1.54(-1.69,-1.39) |
| Cabo Verde | 9.38(5.87,14.91) | 0.25(-0.10,0.61) | 196.05(120.97,304.51) | 0.02(-0.29,0.32) |
| Cambodia | 9.27(6.75,11.88) | 0.37(0.28,0.45) | 216.16(154.83,279.15) | 0.11(0.03,0.19) |
| Cameroon | 6.17(4.14,9.42) | 0.32(0.24,0.39) | 134.88(87.25,204.82) | 0.18(0.10,0.27) |
| Canada | 7.35(5.47,9.04) | -1.06(-1.10,-1.01) | 161.28(121.90,196.90) | -1.27(-1.32,-1.21) |
| Central African Republic | 7.21(4.68,10.67) | -0.51(-0.56,-0.46) | 183.68(116.31,274.72) | -0.56(-0.61,-0.50) |
| Chad | 6.75(4.75,10.51) | 1.24(1.14,1.35) | 150.60(104.32,233.63) | 1.13(1.03,1.24) |
| Chile | 8.78(6.32,12.78) | -0.54(-0.62,-0.46) | 178.75(129.83,259.39) | -0.61(-0.73,-0.50) |
| China | 9.36(6.06,14.59) | -1.33(-1.58,-1.08) | 212.85(137.39,328.40) | -1.50(-1.74,-1.26) |
| Colombia | 4.98(3.06,8.43) | -1.45(-1.58,-1.32) | 114.03(69.15,196.17) | -1.30(-1.45,-1.16) |
| Comoros | 6.50(4.29,9.07) | -0.24(-0.36,-0.12) | 151.04(96.49,213.39) | -0.34(-0.51,-0.17) |
| Congo | 8.71(6.22,11.58) | -0.79(-0.98,-0.59) | 201.63(140.74,272.91) | -1.06(-1.26,-0.85) |
| Cook Islands | 5.14(3.60,7.06) | -0.95(-1.10,-0.80) | 115.86(80.03,160.58) | -0.96(-1.14,-0.77) |
| Costa Rica | 6.87(4.08,11.79) | -0.25(-0.43,-0.06) | 150.53(88.93,256.73) | -0.21(-0.42,0.01) |
| Croatia | 9.66(6.37,13.37) | -0.30(-0.47,-0.13) | 208.25(138.71,292.94) | -0.62(-0.78,-0.45) |
| Cuba | 7.02(4.85,9.11) | -0.41(-0.59,-0.24) | 149.68(103.10,196.47) | -0.53(-0.71,-0.34) |
| Cyprus | 6.71(4.89,8.44) | 0.44(0.22,0.66) | 133.61(98.33,168.30) | 0.28(0.08,0.49) |
| Czechia | 8.96(6.18,12.07) | -1.75(-1.90,-1.59) | 195.63(136.85,262.90) | -1.99(-2.14,-1.85) |
| Côte d'Ivoire | #N/A | #N/A | #N/A | #N/A |
| Democratic People's Republic of Korea | 9.00(5.41,14.86) | -0.04(-0.12,0.04) | 229.83(135.98,382.15) | -0.11(-0.18,-0.04) |
| Democratic Republic of the Congo | 5.90(3.62,8.58) | -0.19(-0.26,-0.12) | 138.80(85.82,197.18) | -0.19(-0.27,-0.10) |
| Denmark | 9.10(6.83,11.06) | -1.48(-1.61,-1.36) | 187.05(141.96,226.72) | -1.88(-2.01,-1.75) |
| Djibouti | 9.90(6.74,14.52) | -0.30(-0.37,-0.22) | 230.31(151.87,349.60) | -0.41(-0.50,-0.32) |
| Dominica | 6.52(4.01,11.13) | 0.18(0.09,0.27) | 139.21(84.28,240.34) | 0.04(-0.07,0.14) |
| Dominican Republic | 5.43(3.64,7.93) | 1.69(1.52,1.86) | 122.88(79.40,181.11) | 1.65(1.51,1.80) |
| Ecuador | 6.31(3.75,11.71) | 0.74(0.47,1.00) | 132.44(76.83,242.66) | 0.62(0.36,0.87) |
| Egypt | 2.79(1.84,3.93) | 0.27(0.16,0.37) | 69.22(44.93,98.67) | 0.19(0.07,0.31) |
| El Salvador | 4.80(2.72,9.01) | 0.47(0.19,0.76) | 110.40(62.77,203.69) | 0.33(0.04,0.61) |
| Equatorial Guinea | 7.51(4.96,10.99) | 0.17(-0.03,0.37) | 164.02(104.05,250.07) | -0.38(-0.65,-0.12) |
| Eritrea | 8.75(5.93,12.25) | -0.01(-0.20,0.17) | 214.63(143.40,305.07) | -0.24(-0.41,-0.07) |
| Estonia | 7.77(5.29,10.42) | -1.03(-1.21,-0.85) | 171.28(115.44,233.36) | -1.63(-1.82,-1.43) |
| Eswatini | 10.57(6.96,14.87) | 0.05(-0.43,0.53) | 255.46(162.51,371.01) | 0.07(-0.49,0.63) |
| Ethiopia | 4.92(3.44,7.21) | -1.19(-1.38,-1.00) | 112.33(78.75,164.48) | -1.66(-1.87,-1.45) |
| Fiji | 6.99(5.13,9.42) | 0.44(0.24,0.64) | 157.04(114.08,212.84) | 0.35(0.13,0.58) |
| Finland | 5.59(4.17,6.97) | -1.17(-1.30,-1.05) | 120.00(89.24,149.10) | -1.30(-1.41,-1.18) |
| France | 8.73(6.67,10.52) | -1.27(-1.33,-1.21) | 190.82(147.99,228.63) | -1.28(-1.35,-1.21) |
| Gabon | 8.42(5.98,10.92) | -0.31(-0.39,-0.22) | 191.85(129.71,256.48) | -0.49(-0.58,-0.39) |
| Gambia | 4.32(3.03,5.86) | 0.61(0.46,0.75) | 95.62(66.47,129.81) | 0.54(0.36,0.72) |
| Georgia | 7.55(5.19,11.06) | 1.05(0.58,1.51) | 194.77(132.81,285.77) | 0.73(0.28,1.18) |
| Germany | 8.27(6.30,10.21) | -1.23(-1.72,-0.74) | 177.55(135.86,218.55) | -1.32(-1.66,-0.98) |
| Ghana | 4.52(3.14,6.72) | 0.13(0.08,0.19) | 99.40(68.98,145.05) | 0.01(-0.06,0.07) |
| Greece | 6.64(4.99,8.46) | -0.38(-0.58,-0.18) | 141.02(105.62,180.89) | -0.27(-0.39,-0.16) |
| Greenland | 14.70(10.65,19.26) | -0.95(-1.22,-0.67) | 330.58(238.75,441.67) | -1.11(-1.35,-0.88) |
| Grenada | 8.45(6.22,10.84) | 0.49(0.37,0.61) | 183.93(136.06,236.49) | 0.38(0.26,0.50) |
| Guam | 6.39(4.59,8.31) | -0.86(-1.22,-0.50) | 153.87(110.77,200.29) | -0.34(-0.66,-0.02) |
| Guatemala | 6.20(3.33,13.03) | 0.15(-0.30,0.59) | 136.08(71.83,287.91) | 0.21(-0.19,0.61) |
| Guinea | 4.58(2.86,8.11) | 0.82(0.76,0.88) | 103.30(63.87,184.70) | 0.86(0.79,0.92) |
| Guinea-Bissau | 7.37(4.82,12.01) | 0.20(0.09,0.32) | 172.81(110.06,273.07) | 0.06(-0.06,0.18) |
| Guyana | 6.13(4.28,8.60) | -0.42(-0.56,-0.29) | 143.73(97.92,204.53) | -0.32(-0.45,-0.18) |
| Haiti | 6.70(4.11,10.88) | -0.05(-0.12,0.03) | 154.69(92.20,250.63) | -0.14(-0.23,-0.04) |
| Honduras | 5.06(2.80,8.93) | 1.28(1.13,1.43) | 111.85(62.03,197.30) | 0.91(0.79,1.02) |
| Hungary | 13.17(9.32,17.26) | -0.52(-0.75,-0.30) | 303.56(218.37,401.51) | -0.55(-0.79,-0.30) |
| Iceland | 6.35(4.75,7.97) | -1.57(-1.69,-1.44) | 137.72(103.29,172.14) | -1.71(-1.81,-1.62) |
| India | 4.65(3.42,6.49) | -0.25(-0.38,-0.12) | 107.93(77.14,154.40) | -0.31(-0.43,-0.19) |
| Indonesia | 8.31(5.60,11.19) | 0.93(0.88,0.98) | 186.19(126.55,251.21) | 0.63(0.56,0.70) |
| Iran (Islamic Republic of) | 4.49(3.30,6.88) | 0.09(-0.15,0.33) | 98.80(72.60,151.41) | -0.08(-0.34,0.17) |
| Iraq | 4.62(3.37,6.00) | 0.73(0.60,0.85) | 108.33(77.54,144.83) | 0.62(0.51,0.73) |
| Ireland | 8.27(6.24,10.23) | -1.68(-1.73,-1.62) | 173.58(132.98,214.75) | -1.91(-1.97,-1.85) |
| Israel | 5.95(4.15,7.64) | -1.69(-1.97,-1.42) | 121.63(86.22,155.37) | -1.65(-1.92,-1.38) |
| Italy | 7.28(5.49,9.30) | -1.24(-1.30,-1.18) | 157.17(119.23,198.90) | -1.39(-1.46,-1.32) |
| Jamaica | 6.98(4.82,9.90) | 1.08(0.78,1.37) | 160.48(109.98,223.86) | 1.30(0.96,1.64) |
| Japan | 8.02(5.70,11.43) | -1.04(-1.09,-0.98) | 174.17(127.82,240.04) | -1.17(-1.23,-1.11) |
| Jordan | 5.95(4.46,7.41) | 0.00(-0.09,0.09) | 129.47(96.38,162.84) | -0.33(-0.40,-0.26) |
| Kazakhstan | 7.92(5.62,11.47) | -2.67(-2.77,-2.56) | 186.69(131.80,271.55) | -2.97(-3.08,-2.85) |
| Kenya | 6.38(4.20,9.23) | 1.08(0.90,1.27) | 147.16(94.63,213.70) | 0.98(0.79,1.17) |
| Kiribati | 8.57(5.59,12.91) | -0.34(-0.42,-0.27) | 209.61(133.98,324.02) | -0.52(-0.60,-0.44) |
| Kuwait | 3.69(2.53,4.74) | 0.99(0.62,1.36) | 74.71(51.73,97.73) | 0.59(0.20,0.99) |
| Kyrgyzstan | 5.49(3.61,9.40) | -2.43(-2.67,-2.20) | 128.86(81.98,228.50) | -3.07(-3.34,-2.79) |
| Lao People's Democratic Republic | 7.74(5.35,10.42) | -0.74(-0.86,-0.62) | 182.23(120.94,249.52) | -1.04(-1.16,-0.92) |
| Latvia | 8.47(5.99,11.38) | -0.86(-1.12,-0.60) | 193.71(133.52,264.72) | -1.31(-1.60,-1.03) |
| Lebanon | 6.62(4.58,8.93) | 1.52(1.30,1.74) | 145.84(100.59,196.12) | 1.43(1.22,1.65) |
| Lesotho | 11.69(7.92,15.99) | 1.57(1.36,1.79) | 288.67(189.97,402.75) | 1.71(1.46,1.97) |
| Liberia | 5.50(3.43,8.60) | 0.60(0.30,0.90) | 119.55(74.33,183.71) | 0.46(0.14,0.78) |
| Libya | 5.35(3.64,7.21) | 0.23(0.11,0.34) | 127.48(85.51,174.36) | 0.16(0.05,0.27) |
| Lithuania | 8.14(5.62,11.00) | -0.73(-0.93,-0.54) | 188.07(128.78,261.55) | -1.08(-1.31,-0.84) |
| Luxembourg | 7.50(5.66,9.42) | -1.98(-2.13,-1.83) | 157.85(119.92,198.57) | -2.11(-2.26,-1.97) |
| Madagascar | 6.47(4.38,9.33) | -0.41(-0.56,-0.25) | 156.92(104.30,226.33) | -0.50(-0.64,-0.36) |
| Malawi | 8.66(4.89,13.09) | 0.08(-0.11,0.27) | 210.15(114.66,325.46) | -0.03(-0.26,0.20) |
| Malaysia | 9.81(7.21,12.84) | -0.42(-0.63,-0.21) | 205.02(148.86,273.00) | -0.54(-0.74,-0.35) |
| Maldives | 4.25(3.06,5.33) | -1.96(-2.18,-1.74) | 83.80(60.49,104.82) | -2.52(-2.79,-2.24) |
| Mali | 5.04(3.18,9.27) | -0.14(-0.22,-0.07) | 116.79(72.16,218.46) | -0.32(-0.42,-0.21) |
| Malta | 6.64(4.99,8.29) | -0.94(-1.01,-0.86) | 142.75(107.53,178.31) | -1.00(-1.07,-0.92) |
| Marshall Islands | 8.83(5.72,13.22) | 0.22(0.15,0.28) | 210.52(132.19,322.31) | 0.21(0.15,0.27) |
| Mauritania | 5.76(3.91,8.61) | -0.02(-0.20,0.16) | 121.53(80.34,184.54) | -0.32(-0.51,-0.12) |
| Mauritius | 5.94(4.09,8.19) | -0.31(-0.39,-0.23) | 134.87(92.32,186.07) | -0.37(-0.45,-0.29) |
| Mexico | 4.05(2.83,6.04) | 0.01(-0.14,0.17) | 95.16(66.48,141.91) | 0.25(0.10,0.39) |
| Micronesia (Federated States of) | 9.39(5.93,13.89) | 0.17(0.13,0.20) | 223.42(136.41,340.56) | 0.10(0.06,0.14) |
| Monaco | 9.60(6.75,12.65) | 0.24(0.13,0.36) | 210.20(147.19,283.16) | 0.16(0.06,0.26) |
| Mongolia | 18.16(11.26,29.52) | -1.31(-1.55,-1.08) | 389.57(240.92,652.87) | -1.57(-1.80,-1.33) |
| Montenegro | 6.76(4.70,9.12) | 0.17(0.05,0.30) | 156.79(110.24,210.65) | -0.06(-0.23,0.12) |
| Morocco | 3.91(2.75,5.21) | -0.03(-0.26,0.19) | 91.96(64.10,123.15) | -0.15(-0.33,0.03) |
| Mozambique | 7.31(5.05,9.84) | 1.29(1.17,1.40) | 165.19(112.69,226.85) | 1.39(1.26,1.52) |
| Myanmar | 7.79(5.54,10.30) | -0.23(-0.27,-0.18) | 182.48(126.93,241.79) | -0.55(-0.61,-0.49) |
| Namibia | 5.40(4.13,7.08) | 0.88(0.76,1.01) | 124.08(92.68,168.46) | 0.68(0.50,0.86) |
| Nauru | 10.43(6.68,15.13) | -0.23(-0.32,-0.15) | 250.01(155.61,364.12) | -0.27(-0.39,-0.14) |
| Nepal | 4.42(2.77,6.73) | 0.23(-0.05,0.51) | 99.88(62.65,154.64) | -0.13(-0.45,0.18) |
| Netherlands | 9.79(7.38,12.07) | -0.36(-0.46,-0.26) | 206.26(156.87,249.81) | -0.61(-0.69,-0.54) |
| New Zealand | 10.04(7.95,11.86) | -1.16(-1.23,-1.09) | 218.20(174.82,257.85) | -1.42(-1.50,-1.35) |
| Nicaragua | 5.88(3.91,9.61) | 0.73(0.47,0.99) | 120.60(77.86,197.85) | 0.41(0.24,0.58) |
| Niger | 4.85(3.09,8.31) | 0.03(-0.10,0.17) | 105.91(66.74,179.74) | -0.23(-0.39,-0.08) |
| Nigeria | 4.27(3.17,5.58) | 1.05(0.89,1.20) | 87.99(63.68,118.18) | 0.88(0.74,1.02) |
| Niue | 7.98(5.56,11.07) | -0.13(-0.18,-0.09) | 180.86(121.25,251.86) | -0.24(-0.28,-0.19) |
| North Macedonia | 9.61(6.27,14.30) | 0.39(0.17,0.60) | 217.97(141.57,328.18) | 0.05(-0.14,0.25) |
| Northern Mariana Islands | 9.59(6.99,12.63) | -0.19(-0.32,-0.06) | 211.30(151.05,277.33) | -0.26(-0.38,-0.14) |
| Norway | 8.31(6.31,10.06) | -0.68(-0.80,-0.57) | 170.09(130.32,204.79) | -0.95(-1.08,-0.82) |
| Oman | 3.70(2.56,5.00) | 0.36(0.23,0.49) | 73.33(49.44,99.84) | -0.14(-0.36,0.08) |
| Pakistan | 7.34(5.38,9.68) | 0.78(0.56,1.01) | 181.15(130.53,239.95) | 0.78(0.53,1.03) |
| Palau | 8.46(5.72,11.44) | -0.20(-0.25,-0.15) | 189.19(125.01,263.05) | -0.22(-0.28,-0.17) |
| Palestine | 10.03(8.05,12.12) | 0.80(0.55,1.05) | 222.71(177.42,271.54) | 0.60(0.35,0.85) |
| Panama | 5.51(3.60,8.68) | -0.27(-0.33,-0.20) | 122.57(79.56,192.41) | -0.36(-0.44,-0.27) |
| Papua New Guinea | 6.14(3.96,9.83) | 0.37(0.34,0.39) | 147.02(94.41,237.79) | 0.35(0.32,0.38) |
| Paraguay | 6.24(4.29,9.06) | 1.34(1.10,1.58) | 142.10(96.09,207.51) | 1.26(1.03,1.50) |
| Peru | 4.91(2.79,9.52) | -0.85(-1.03,-0.68) | 104.74(58.48,204.88) | -1.03(-1.20,-0.86) |
| Philippines | 7.76(5.99,9.86) | -0.17(-0.41,0.08) | 192.84(149.32,246.41) | -0.12(-0.35,0.11) |
| Poland | 11.23(7.95,14.65) | -0.33(-0.46,-0.19) | 248.47(176.28,322.71) | -0.55(-0.68,-0.42) |
| Portugal | 8.68(6.41,11.42) | -0.45(-0.62,-0.27) | 193.61(142.88,254.26) | -0.44(-0.65,-0.23) |
| Puerto Rico | 5.83(3.89,8.04) | -1.03(-1.14,-0.91) | 133.04(88.94,184.22) | -0.90(-1.01,-0.79) |
| Qatar | 5.11(3.32,6.91) | 0.44(0.18,0.71) | 88.87(56.58,120.76) | -0.14(-0.40,0.12) |
| Republic of Korea | 7.42(5.26,10.90) | -1.59(-1.76,-1.42) | 149.01(104.34,221.90) | -2.16(-2.33,-1.99) |
| Republic of Moldova | 7.20(5.13,9.50) | 0.03(-0.43,0.49) | 182.74(130.03,243.62) | -0.19(-0.63,0.25) |
| Romania | 8.43(5.80,11.40) | 0.64(0.46,0.81) | 208.35(142.63,282.87) | 0.39(0.21,0.57) |
| Russian Federation | 8.67(6.16,11.82) | -1.52(-1.81,-1.23) | 204.60(144.65,280.32) | -1.84(-2.16,-1.51) |
| Rwanda | 4.95(3.31,7.01) | -1.75(-2.02,-1.47) | 112.71(73.60,163.53) | -2.24(-2.58,-1.90) |
| Saint Kitts and Nevis | 9.72(7.38,12.49) | -0.49(-0.67,-0.32) | 210.27(155.31,275.77) | -0.91(-1.12,-0.71) |
| Saint Lucia | 6.54(4.65,9.38) | -0.70(-0.98,-0.41) | 144.83(102.71,207.99) | -0.58(-0.83,-0.33) |
| Saint Vincent and the Grenadines | 6.53(4.69,9.14) | -0.45(-0.73,-0.18) | 143.56(101.93,201.33) | -0.53(-0.79,-0.27) |
| Samoa | 5.60(4.01,8.23) | -0.41(-0.46,-0.36) | 129.80(92.57,187.75) | -0.44(-0.49,-0.39) |
| San Marino | 9.38(5.48,15.18) | -0.33(-0.45,-0.22) | 192.95(111.14,318.32) | -0.32(-0.44,-0.20) |
| Sao Tome and Principe | 8.16(5.41,12.36) | 1.02(0.94,1.10) | 169.20(111.37,255.65) | 0.87(0.79,0.95) |
| Saudi Arabia | 3.66(2.57,4.76) | 0.60(0.36,0.85) | 84.27(57.91,111.84) | 0.75(0.54,0.96) |
| Senegal | 6.53(4.51,9.89) | 0.45(0.22,0.67) | 141.65(95.28,213.75) | 0.29(0.04,0.55) |
| Serbia | 10.16(6.88,13.52) | 0.17(0.01,0.33) | 229.12(155.43,311.50) | -0.17(-0.35,0.01) |
| Seychelles | 10.63(8.18,13.13) | 0.29(0.12,0.45) | 234.11(179.06,291.14) | 0.02(-0.13,0.17) |
| Sierra Leone | 5.65(3.71,8.96) | 0.59(0.43,0.76) | 123.75(80.79,194.72) | 0.53(0.35,0.71) |
| Singapore | 5.85(4.28,7.41) | -2.75(-2.85,-2.65) | 120.56(89.40,151.79) | -2.96(-3.05,-2.87) |
| Slovakia | 12.18(8.62,16.23) | -0.14(-0.31,0.03) | 273.28(191.78,369.94) | -0.47(-0.62,-0.31) |
| Slovenia | 8.26(5.61,11.58) | -1.20(-1.40,-0.99) | 176.93(119.13,249.71) | -1.50(-1.71,-1.29) |
| Solomon Islands | 8.63(5.27,14.66) | 0.28(0.21,0.34) | 224.15(129.07,388.38) | 0.30(0.23,0.37) |
| Somalia | 7.91(4.57,12.69) | -0.54(-0.59,-0.49) | 198.44(112.99,319.05) | -0.65(-0.70,-0.59) |
| South Africa | 8.88(7.02,10.98) | -0.66(-1.10,-0.23) | 200.07(156.91,246.77) | -0.98(-1.42,-0.54) |
| South Sudan | 6.49(4.10,9.65) | -0.66(-0.70,-0.62) | 148.33(90.34,229.51) | -0.80(-0.86,-0.74) |
| Spain | 7.79(5.91,9.57) | -0.28(-0.35,-0.21) | 168.06(128.64,205.74) | -0.50(-0.57,-0.43) |
| Sri Lanka | 4.40(3.01,6.32) | 0.34(0.11,0.57) | 96.23(64.88,139.25) | 0.12(-0.12,0.35) |
| Sudan | 4.70(3.01,7.56) | 0.47(0.41,0.53) | 108.90(68.01,180.45) | 0.29(0.23,0.35) |
| Suriname | 7.45(5.58,9.67) | 0.70(0.44,0.97) | 168.94(126.64,220.57) | 0.66(0.41,0.90) |
| Sweden | 6.75(5.11,8.11) | -0.80(-0.90,-0.70) | 141.20(108.06,169.15) | -0.93(-1.03,-0.82) |
| Switzerland | 5.97(4.53,7.29) | -1.18(-1.24,-1.11) | 129.34(99.24,157.14) | -1.43(-1.50,-1.37) |
| Syrian Arab Republic | 2.98(2.04,4.13) | 0.29(0.06,0.52) | 66.73(44.56,94.93) | -0.05(-0.32,0.22) |
| Taiwan (Province of China) | 11.20(7.59,15.36) | 1.21(1.01,1.40) | 256.39(174.58,354.71) | 1.10(0.90,1.29) |
| Tajikistan | 8.35(5.32,14.37) | -0.25(-0.45,-0.04) | 187.41(117.50,326.34) | -1.11(-1.30,-0.92) |
| Thailand | 5.60(3.94,7.84) | -1.60(-1.82,-1.38) | 130.15(90.07,184.77) | -1.80(-2.07,-1.53) |
| Timor-Leste | 7.79(5.30,10.54) | 1.13(0.89,1.36) | 177.29(116.21,241.52) | 0.90(0.63,1.16) |
| Togo | 6.55(4.39,9.86) | 0.48(0.41,0.55) | 146.67(96.68,220.30) | 0.41(0.34,0.47) |
| Tokelau | 6.86(4.73,9.75) | -0.10(-0.11,-0.09) | 156.07(106.04,227.07) | -0.19(-0.20,-0.17) |
| Tonga | 6.04(3.97,9.69) | -0.07(-0.24,0.09) | 134.23(89.01,216.17) | -0.09(-0.25,0.06) |
| Trinidad and Tobago | 5.75(3.87,7.91) | -1.25(-1.40,-1.09) | 130.89(87.79,183.23) | -1.12(-1.29,-0.96) |
| Tunisia | 3.84(2.49,5.41) | -0.35(-0.43,-0.27) | 83.76(54.12,118.97) | -0.36(-0.43,-0.29) |
| Turkey | 4.53(3.03,6.32) | -0.23(-0.71,0.25) | 101.24(66.69,143.65) | -0.72(-1.20,-0.24) |
| Turkmenistan | 4.70(3.00,7.11) | -3.23(-3.76,-2.69) | 117.81(74.60,179.40) | -3.39(-3.97,-2.80) |
| Tuvalu | 7.69(4.94,11.35) | -0.23(-0.26,-0.19) | 179.83(114.40,269.06) | -0.32(-0.35,-0.28) |
| Uganda | 7.01(4.70,9.49) | 1.03(0.84,1.22) | 164.91(108.60,224.56) | 0.98(0.76,1.21) |
| Ukraine | 8.48(5.90,11.70) | -2.11(-2.52,-1.70) | 222.98(152.42,313.61) | -2.28(-2.75,-1.82) |
| United Arab Emirates | 6.76(4.19,9.68) | 0.17(-0.22,0.57) | 131.29(81.10,188.11) | 0.05(-0.26,0.37) |
| United Kingdom | 9.18(7.11,11.15) | -1.48(-1.63,-1.32) | 189.41(147.13,227.72) | -1.68(-1.84,-1.53) |
| United Republic of Tanzania | 6.84(4.54,9.66) | -0.90(-0.99,-0.81) | 158.73(102.14,226.99) | -1.03(-1.12,-0.93) |
| United States of America | 7.27(5.66,8.69) | -1.29(-1.37,-1.21) | 168.86(132.13,200.97) | -1.27(-1.37,-1.18) |
| United States Virgin Islands | 9.55(6.34,12.45) | 0.71(0.53,0.89) | 207.70(136.89,275.85) | 0.68(0.50,0.86) |
| Uruguay | 13.11(9.87,16.30) | -1.05(-1.12,-0.98) | 281.20(214.26,347.27) | -1.21(-1.27,-1.14) |
| Uzbekistan | 5.98(4.04,8.99) | -1.48(-1.68,-1.28) | 136.09(88.88,213.56) | -2.29(-2.57,-2.00) |
| Vanuatu | 6.99(4.46,11.00) | 0.38(0.25,0.51) | 166.65(104.13,262.79) | 0.41(0.26,0.57) |
| Venezuela (Bolivarian Republic of) | 6.13(4.00,9.54) | -0.18(-0.34,-0.02) | 142.30(92.46,220.85) | -0.14(-0.29,0.01) |
| Viet Nam | 10.15(7.31,13.11) | 1.12(1.07,1.17) | 242.57(171.99,321.88) | 1.12(1.03,1.20) |
| Yemen | 5.48(3.53,8.85) | 0.44(0.39,0.48) | 129.59(81.74,209.77) | 0.30(0.26,0.35) |
| Zambia | 11.81(8.26,15.65) | -0.49(-0.66,-0.33) | 286.63(197.83,387.76) | -0.58(-0.77,-0.38) |
| Zimbabwe | 12.74(8.90,16.94) | 0.46(0.24,0.67) | 307.33(208.46,411.41) | 0.64(0.39,0.89) |
